# Reporting recommendations for tumor marker prognostic studies (REMARK): explanation and elaboration

**DOI:** 10.1186/1741-7015-10-51

**Published:** 2012-05-29

**Authors:** Douglas G Altman, Lisa M McShane, Willi Sauerbrei, Sheila E Taube

**Affiliations:** 1 Centre for Statistics in Medicine, University of Oxford, Oxford, OX2 6UD, UK; 2 US National Cancer Institute, Bethesda, MD 20892, USA; 3 Institut fuer Medizinische Biometrie und Medizinische Informatik, Universitaetsklinikum Freiburg, 79104 Freiburg, Germany; 4 ST-Consulting, Bethesda, MD 20817, USA

## Abstract

**Background:**

The Reporting Recommendations for Tumor Marker Prognostic Studies (REMARK) checklist consists of 20 items to report for published tumor marker prognostic studies. It was developed to address widespread deficiencies in the reporting of such studies. In this paper we expand on the REMARK checklist to enhance its use and effectiveness through better understanding of the intent of each item and why the information is important to report.

**Methods:**

REMARK recommends including a transparent and full description of research goals and hypotheses, subject selection, specimen and assay considerations, marker measurement methods, statistical design and analysis, and study results. Each checklist item is explained and accompanied by published examples of good reporting, and relevant empirical evidence of the quality of reporting. We give prominence to discussion of the 'REMARK profile', a suggested tabular format for summarizing key study details.

**Summary:**

The paper provides a comprehensive overview to educate on good reporting and provide a valuable reference for the many issues to consider when designing, conducting, and analyzing tumor marker studies and prognostic studies in medicine in general.

To encourage dissemination of the Reporting Recommendations for Tumor Marker Prognostic Studies (REMARK): Explanation and Elaboration, this article has also been published in PLoS Medicine.

## Background

The purpose of this paper is to provide more complete explanations of each of the Reporting Recommendations for Tumor Marker Prognostic Studies (REMARK) checklist items and to provide specific examples of good reporting drawn from the published literature. The initial REMARK paper [1-7] recommended items that should be reported in all published tumor marker prognostic studies (Table 1). The recommendations were developed by a committee initially convened under the auspices of the National Cancer Institute and the European Organisation for Research and Treatment of Cancer. They were based on the rationale that more transparent and complete reporting of studies would enable others to better judge the usefulness of the data and to interpret the study results in the appropriate context. Similar explanation and elaboration papers had been written to accompany other reporting guidelines [8-11]. No changes to the REMARK checklist items are being suggested here. We hope that the current paper will serve an educational role and lead to more effective implementation of the REMARK recommendations, resulting in more consistent, high quality reporting of tumor marker studies.

**Table 1:** The REMARK checklist [1-7]

**INTRODUCTION**	
1	State the marker examined, the study objectives, and any pre-specified hypotheses.
**MATERIALS AND METHODS**	
*Patients*	
2	Describe the characteristics (for example, disease stage or co-morbidities) of the study patients, including their source and inclusion and exclusion criteria.
3	Describe treatments received and how chosen (for example, randomized or rule-based).
*Specimen characteristics*	
4	Describe type of biological material used (including control samples) and methods of preservation and storage.
*Assay methods*	
5	Specify the assay method used and provide (or reference) a detailed protocol, including specific reagents or kits used, quality control procedures, reproducibility assessments, quantitation methods, and scoring and reporting protocols. Specify whether and how assays were performed blinded to the study endpoint.
*Study design*	
6	State the method of case selection, including whether prospective or retrospective and whether stratification or matching (for example, by stage of disease or age) was used. Specify the time period from which cases were taken, the end of the follow-up period, and the median follow-up time.
7	Precisely define all clinical endpoints examined.
8	List all candidate variables initially examined or considered for inclusion in models.
9	Give rationale for sample size; if the study was designed to detect a specified effect size, give the target power and effect size.
*Statistical analysis methods*	
10	Specify all statistical methods, including details of any variable selection procedures and other model-building issues, how model assumptions were verified, and how missing data were handled.
11	Clarify how marker values were handled in the analyses; if relevant, describe methods used for cutpoint determination.
**RESULTS**	
*Data *	
12	Describe the flow of patients through the study, including the number of patients included in each stage of the analysis (a diagram may be helpful) and reasons for dropout. Specifically, both overall and for each subgroup extensively examined report the number of patients and the number of events.
13	Report distributions of basic demographic characteristics (at least age and sex), standard (disease-specific) prognostic variables, and tumor marker, including numbers of missing values.
*Analysis and presentation *	
14	Show the relation of the marker to standard prognostic variables.
15	Present univariable analyses showing the relation between the marker and outcome, with the estimated effect (for example, hazard ratio and survival probability). Preferably provide similar analyses for all other variables being analyzed. For the effect of a tumor marker on a time-to-event outcome, a Kaplan-Meier plot is recommended.
16	For key multivariable analyses, report estimated effects (for example, hazard ratio) with confidence intervals for the marker and, at least for the final model, all other variables in the model.
17	Among reported results, provide estimated effects with confidence intervals from an analysis in which the marker and standard prognostic variables are included, regardless of their statistical significance.
18	If done, report results of further investigations, such as checking assumptions, sensitivity analyses, and internal validation.
**DISCUSSION**	
19	Interpret the results in the context of the pre-specified hypotheses and other relevant studies; include a discussion of limitations of the study.
20	Discuss implications for future research and clinical value.

we have changed 'univariate' to 'univariable' in item 15 for consistency with 'multivariable'.

Our intent is to explain how to properly report prognostic marker research, not to specify how to perform the research. However, we believe that fundamental to an appreciation of the importance of good reporting is a basic understanding of how various factors such as specimen selection, marker assay methodology and statistical study design and analysis can lead to different study results and interpretations. Many authors have discussed the fact that widespread methodological and reporting deficiencies plague the prognostic literature in cancer and other specialties [12-21]. Careful reporting of what was done and what results were obtained allows for better assessment of study quality and greater understanding of the relevance of the study conclusions. When available, we have cited published studies presenting empirical evidence of the quality of reporting of the information requested by the checklist items.

We recognize that tumor marker studies are generally collaborative efforts among researchers from a variety of disciplines. The current paper covers a wide range of topics and readers representing different disciplines may find certain parts of the paper more accessible than other parts. Nonetheless, it is helpful if all involved have a basic understanding of the collective obligations of the study team.

We have attempted to minimize distractions from more highly technical material by the use of boxes with supplementary information. The boxes are intended to help readers refresh their memories about some theoretical points or be quickly informed about technical background details. A full understanding of these points may require studying the cited references.

We aimed to provide a comprehensive overview that not only educates on good reporting but provides a valuable reference for the many issues to consider when designing, conducting and analyzing tumor marker studies. Each item is accompanied by one or more examples of good reporting drawn from the published literature. We hope that readers will find the paper useful not only when they are reporting their studies but also when they are planning their studies and analyzing their study data.

This paper is structured as the original checklist, according to the typical sections of scientific reports: Introduction, Materials and Methods, Results and Discussion. There are numerous instances of cross-referencing between sections reflecting the fact that the sections are inter-related, for example, one must speak about the analysis methods used in order to discuss presentation of results obtained using those methods. These cross-references do not represent redundancies in the material presented and readers are reminded that distinctions in focus and emphasis between different items will sometimes be subtle.

One suggestion in the REMARK checklist is to include a diagram showing the flow of patients through the study (see Item 12). We elaborate upon that idea in the current paper. The flow diagram is an important element of the Consolidated Standards of Reporting Trials (CONSORT) Statement, which was developed to improve reporting of randomized controlled trials (RCTs) [8,22,23]. Many papers reporting randomized trial results present a flow diagram showing numbers of patients registered and randomized, numbers of patients excluded or lost to follow-up by treatment arms, and numbers analyzed. Flow diagrams are also recommended in the Strengthening the Reporting of Observational Studies in Epidemiology (STROBE) Statement for reporting observational studies, including cohort studies [9]. A diagram would indeed be useful for prognostic studies to clarify the numbers and characteristics of patients included at each stage of the study. There are additional key aspects of prognostic studies that need to be reported and would benefit from standardized presentation. Accordingly we have developed a 'REMARK profile' as a proposed format for describing succinctly key aspects of the design and analysis of a prognostic marker study; we discuss the profile in detail in Item 12 below.

The original scope of the REMARK recommendations focused on studies of prognostic tumor markers that reported measurement of biological molecules found in tissues, blood and other body fluids. The recommendations also apply more generally to prognostic factors other than biological molecules that are often assessed in cancer patients, including the size of the tumor, abnormal features of the cells, the presence of tumor cells in regional lymph nodes, age and gender among others. Prognostic research includes study of the wide variety of indicators that help clinicians predict the course of a patient's disease in the context of standard care. REMARK generally applies to any studies involving prognostic factors, whether those prognostic factors are biological markers, imaging assessments, clinical assessments or measures of functional status in activities of daily living. REMARK applies to other diseases in addition to cancer. The processes of measuring and reporting the prognostic factors may differ, but the same study reporting principles apply.

We suggest that most of the recommendations also apply to studies looking at the usefulness of a marker for the prediction of benefit from therapy (typically called a predictive marker in oncology). Traditionally, predictive markers are evaluated by determination of whether the benefit of the treatment of interest compared to another standard treatment depends on the marker status or value. (See also Items 3 and 9 and Box 1 below.) A logical corollary to such a finding is that the prognostic value of that marker depends on the treatment the patient receives; for this reason, some view predictive markers as a special class of prognostic markers. Consequently, REMARK items apply to many aspects of these studies. In the explanations that follow for each of the checklist items, we attempted to make note of some special considerations for studies evaluating predictive markers. We hope that authors who report predictive marker studies will therefore find our recommendations useful. As predictive markers are usually evaluated in randomized trials, CONSORT [11] will also apply to reporting of predictive marker studies.

Although REMARK was primarily aimed at the reporting of studies that have evaluated the prognostic value of a single marker, the recommendations are substantially relevant to studies investigating more than one marker, including studies investigating complex markers that are composed of a few to many components, such as multivariable classification functions or indices, or are based on prognostic decision algorithms. These reporting recommendations do not attempt to address reporting of all aspects of the development or validation of these complex markers, but several key elements of REMARK do also apply to these developmental studies. Moreover, once these complex markers are fully defined, their evaluation in clinical studies is entirely within the scope of REMARK.

The development of prognostic markers generally involves a series of studies. These begin with identification of a relationship between a biological feature (for example, proliferative index or genetic alteration) and a clinical characteristic or outcome. To establish a clear and possibly causal relationship, a series of studies are conducted to address increasingly demanding hypotheses. The REMARK recommendations attempt to recognize these stages of development. For example, the discussion of Item 9 acknowledges that sample size determination may not be under the investigator's control but recommends that authors make clear whether there was a calculated sample size or, if not, consider the impact of the sample size on the reliability of the findings or precision of estimated effects. We anticipate that more details will be available in later stage studies, but many of the recommendations are also applicable to earlier stage studies. When specific items of information recommended by REMARK are not available, these situations should be fully acknowledged in the report so that readers may judge in context whether these missing elements are critical to study interpretation. Adherence to these reporting recommendations as much as possible will permit critical evaluation of the full body of evidence supporting a marker.

## Checklist items

Discussion and explanation of the 20 items in the REMARK checklist (Table 1) are presented. For clarity we have split the discussion of a few items into multiple parts. Each explanation is preceded by examples from the published literature that illustrate types of information that are appropriate to address the item. Our use of an example from a study does not imply that all aspects of the study were well reported or appropriately conducted. The example suggests only that this particular item, or a relevant part of it, was well reported in that study. Some of the quoted examples have been edited by removing citations or spelling out abbreviations, and some tables have been simplified.

Each checklist item should be addressed somewhere in a report even if it can only be addressed by an acknowledgment that the information is unknown. We do not prescribe a precise location or order of presentation as this may be dependent upon journal policies and is best left to the discretion of the authors of the report. We recognize that authors may address several items in a single section of text or in a table. In the current paper, we address reporting of results under a number of separate items to allow us to explain them clearly and provide examples, not to prescribe a heading or location. Authors may find it convenient to report some of the requested items in a supplementary material section, for example on a journal website, rather than in the body of the manuscript to allow sufficient space for adequate detail to be provided. One strategy that has been used successfully is to provide the information in a supplementary table organized according to the order of the REMARK items [24]. The elements of the supplementary table may either provide the information directly in succinct form or point the reader to the relevant section of the main paper where the information can be found. Authors wishing to supply such a supplementary table with their paper may find it helpful to use the REMARK reporting template that is supplied as Additional file 1; it can also be downloaded from http://www.equator-network.org/resource-centre/library-of-health-research-reporting/reporting-guidelines/remark.

## Introduction

### 

#### Item 1. State the marker examined, the study objectives, and any pre-specified hypotheses

##### Examples

###### Marker examined

'Using the same cohort of patients, we investigated the relationship between the type, density, and location of immune cells within tumors and the clinical outcome of the patients.' [25]

###### Objectives

'The purpose of this study was to determine whether CpG island hypermethylation in the promoter region of the APC gene occurs in primary esophageal carcinomas and premalignant lesions, whether freely circulating hypermethylated APC DNA is detectable in the plasma of these patients, and whether the presence and quantity of hypermethylated APC in the plasma have any relationship with outcome.' [26]

'The goal of this study was to develop a sensitive and specific method for CTC [circulating tumor cell] detection in HER-2-positive breast cancer, and to validate its ability to track disease response and progression during therapy.' [27]

###### Hypotheses

"The prespecified hypotheses tested were that TS expression level and p53 expression status are markers of overall survival (OS) in potentially curatively resected CRC.' [28]

##### Explanation

Clear indication of the particular markers to be examined, the study objectives and any pre-specified hypotheses should be provided early in the study report. Objectives are goals one hopes to accomplish by conducting the study. Typical objectives for tumor marker prognostic studies include, among others, an evaluation of the association between tumor marker value and clinical outcome, or determination of whether a tumor marker contributes additional information about likely clinical outcome beyond the information provided by standard clinical or pathologic factors.

The description of the marker should include both the biological aspects of the marker as well as the time in a patient's clinical course when it is to be assessed. The biological aspects should include the type of molecule or structure examined (for example, protein, RNA, DNA or chromosomes) and the features assessed (for example, expression level, copy number, mutation or translocation). Most prognostic marker studies are performed on specimens obtained at the time of initial diagnosis. The marker could also be assessed on specimens collected at completion of an initial course of therapy (for example, detection of minimal residual disease or circulating tumor cells to predict recurrence or progression) or at the time of recurrence or progression. A thorough description of the marker and timing of specimen collection is necessary for an understanding of the biological rationale and potential clinical application.

The stated objectives often lead to the development of specific hypotheses. Hypotheses should be formulated in terms of measures that are amenable to statistical evaluation. They represent tentative assumptions that can be supported or refuted by the results of the study. An example of a hypothesis is 'high expression levels of the protein measured in the tumor at the time of diagnosis are associated with shorter disease-free survival'.

Pre-specified hypotheses are those that are based on prior research or an understanding of a biological mechanism, and they are stated before the study is initiated. Ideally, a systematic review of the literature should have been performed. New hypotheses may be suggested by inspection of data generated in the study. Analyses performed to address the new hypotheses are exploratory and should be reported as such. The distinction between analysis of the pre-specified hypotheses and exploratory analyses is important because it affects the interpretation (see Item 19) [9].

## Materials and methods

### Patients

#### Item 2. Describe the characteristics (for example, disease stage or co-morbidities) of the study patients, including their source and inclusion and exclusion criteria

##### Examples

'Inclusion criteria for the 2810 patients from whom tumour or cytosol samples were stored in our tumour bank (liquid nitrogen) were: primary diagnosis of breast cancer between 1978 and 1992 (at least 5 years of potential follow-up); no metastatic disease at diagnosis; no previous diagnosis of carcinoma, with the exception of basal cell skin carcinoma and cervical cancer stage I; no evidence of disease within 1 month of primary surgery ... Patients with inoperable T4 tumours and patients who received neoadjuvant treatment before primary surgery were excluded." [29]

'We studied 196 adults who were younger than 60 years and who had untreated primary CN-AML. The diagnosis of CN-AML was based on standard cytogenetic analysis that was performed by CALGB-approved institutional cytogenetic laboratories as part of the cytogenetic companion study 8461. To be considered cytogenetically normal, at least 20 metaphase cells from diagnostic bone marrow (BM) had to be evaluated, and the karyotype had to be found normal in each patient. All cytogenetic results were confirmed by central karyotype review. All patients were enrolled on two similar CALGB treatment protocols (i.e., 9621 or 19808).' [30]

'These analyses were conducted within the context of a completed clinical trial for breast cancer (S8897), which was led by SWOG within the North American Breast Cancer Intergroup (INT0102) ... Complete details of S8897 have been reported elsewhere [citation].' [31]

Relevant text in the reference cited by Choi et al. [31]:

'Patients were registered from the Southwest Oncology Group, Eastern Cooperative Oncology Group, and Cancer and Leukemia Group B ... Eligible patients included premenopausal and postmenopausal women with T1 to T3a node negative invasive adenocarcinoma of the breast.' [32]

##### Explanation

Each prognostic factor study includes data from patients drawn from a specific population. A description of that population is needed to place the study in a clinical context. The source of the patients should be specified, for example from a clinical trial population, a healthcare system, a clinical practice or all hospitals in a certain geographic area.

Patient eligibility criteria, usually based on clinical or pathologic characteristics, should be clearly stated. As a minimum, eligibility criteria should specify the site and stage of cancer of the cases to be studied. Stage is particularly important because many tumor markers have prognostic value in early stage disease but not in advanced stage disease. For example, if a marker is indicative of metastatic potential, it may have strong prognostic value in patients with early stage disease but be less informative for patients who already have advanced or metastatic disease. For this reason, many studies are restricted to certain stages. Additional selection criteria may relate to factors such as patient age, treatment received (see Item 3) or the histologic type of cancer.

Exclusion criteria might be factors such as prior cancer, prior systemic treatment for cancer, nonstandard treatment (for example, rarely used, non-approved or 'off-label' use of a therapy), failure to obtain informed consent, insufficient tumor specimen or a high proportion of missing critical clinical or pathologic data. It is generally not appropriate to exclude a case just because it has a few missing data elements if those data elements are not critical for assessment of primary inclusion or exclusion criteria (see Item 6a) [33]. In some studies, deaths that have occurred very early after the initiation of follow-up are excluded. If this is done, the rationale and timeframe for exclusion should be specified. To the extent possible, exclusion criteria should be specified prior to initiation of the study to avoid potential bias introduced by exclusions that could be partly motivated by intermediate analysis results.

When a prognostic study is performed using a subset of cases from a prior 'parent' study (for example, from a RCT or a large observational study cohort), there may be a prior publication or other publicly available document such as a study protocol that lists detailed eligibility and inclusion and exclusion criteria for the parent study. In these cases, the prior document can be referenced rather than repeating all of the details in the prognostic study paper. However, it is preferable that at least the major criteria (for example, the site and stage of the cancer) for the parent study still be mentioned in the prognostic study paper, and it is essential that any additional criteria imposed specifically for the prognostic study (such as availability of adequate specimens) be stated in the prognostic study paper.

Specification of inclusion and exclusion criteria can be especially challenging when the study is conducted retrospectively. The real population that the cases represent is often unclear if the starting point is all cases with accessible medical records or all cases with specimens included in a tumor bank. A review of 96 prognostic studies found that 40 had the availability of tumor specimens or data as an inclusion criterion [33]. In some studies, unknown characteristics may have governed whether cases were represented in the medical record system or tumor bank, making it impossible to specify exact inclusion and exclusion criteria. If the specimen set was assembled primarily on the basis of ready availability (that is, a 'convenience' sample), this should be acknowledged.

A flow diagram is very useful for succinctly describing the characteristics of the study patients. The entrance point to the flow diagram is the source of patients and successive steps in the diagram can represent inclusion and exclusion criteria. Some of the information from this diagram can also be given in the upper part of the REMARK profile (see Item 12 for examples).

After the study population has been defined, it is important to describe how the specific cases included in the study were sampled from that population. Item 6a discusses reporting of case selection methods.

#### Item 3. Describe treatments received and how chosen (for example, randomized or rule-based)

##### Examples

'Patients were treated with surgery by either modified radical mastectomy (637 cases) or local tumour resection (683 cases), with axillary node dissection followed by postoperative breast irradiation (695 cases). Adjuvant therapy with chemotherapy and/or hormone therapy was decided according to nodal status and hormone receptor results. Treatment protocols varied over time. From 1975 to 1985, node-negative patients had no chemotherapy. After 1985, node-negative patients under 50 years of age, with ER and PR negative and SBR [Scarff-Bloom-Richardson] grade 3 tumours, had chemotherapy.' [34]

'Details of the treatment protocols have been previously reported. Briefly, patients on CALGB 9621 received induction chemotherapy with cytarabine, daunorubicin, and etoposide with (ADEP) or without (ADE) the multidrug resistance protein modulator PSC-833, also called valspodar. Patients who had CN-AML and who achieved a CR received high-dose cytarabine (HiDAC) and etoposide for stem-cell mobilization followed by myeloablative treatment with busulfan and etoposide supported by APBSCT. Patients unable to receive APBSCT received two additional cycles of Hi-DAC. Patients enrolled on CALGB 19808 were treated similarly to those on CALGB 9621. None of the patients received allogeneic stem-cell transplantation in first remission.' [30]

##### Explanation

A patient's disease-related clinical outcome is determined by a combination of the inherent biological aggressiveness of a patient's tumor and the response to any therapies received. The influence of biological characteristics on disease outcome would ideally be assessed in patients who received no treatment, but usually most patients will have received some therapy. Many patients with solid tumors will receive local-regional therapy (for example, surgery and possibly radiotherapy). For some types and stages of cancer, patients would almost always receive systemic therapy (for example, chemotherapy or endocrine therapy). Sometimes all patients included in a study will have received a standardized therapy, but more often there will be a mix of treatments that patients have received. The varied treatments that patients might receive in standard care settings can make study of prognostic markers especially challenging.

Because different treatments might alter the disease course in different ways, it is important to report what treatments the patients received. The impact of a treatment might also depend on the biological characteristics of the tumor. This is the essence of predictive marker research where the goal is to identify the treatment that leads to the best clinical outcome for each biological class of tumor (for example, defined by markers) (see Box 1).

The basis for treatment selection, if known, should be reported. If not known, as will often be the case for retrospective specimen collections, one must be cautious in interpreting prognostic and predictive analyses. This concern derives from the possibility that the value of the marker or patient characteristics associated with the marker played a role in the choice of therapy, thereby leading to a potential confounding of effects of treatment and marker. If sufficient numbers of patients are treated with certain therapies, assessment of the prognostic value of the marker separately by treatment group (see Box 1) could be considered. However, predictive markers should generally be evaluated in randomized clinical trials to ensure that the choice of treatment was not influenced by the marker or other biological characteristics of the tumor.

It is also important to report the timing of therapy relative to specimen collection since biological characteristics of a tumor may be altered by the therapies to which it was exposed prior to specimen collection (see Item 4). The prognostic value of a marker may be different depending on whether it was present in the tumor at the time of initial diagnosis, was present only after the patient received therapy or whether it is in the presence of other biological characteristics that emerged as a consequence of therapy.

### Specimen characteristics

#### Item 4. Describe type of biological material used (including control samples) and preservation and storage methods

##### Examples

###### Positive and negative controls

'Tumor specimens were obtained at the time of surgery and snap frozen in liquid nitrogen, then stored at -80°C. Blood samples were collected 24 hours or less before surgery by peripheral venous puncture and were centrifuged at 1500 × g at 4°C for 10 minutes. The separated plasma was aliquoted and stored at -80°C for future analysis. Normal endometrial tissue specimens were obtained from patients undergoing hysterectomy for benign gynecologic pathologies. Control plasma specimens were derived from health check examinees at Yongdong Severance Hospital who showed no history of cancer or gynecologic disease and had no abnormalities in laboratory examinations or gynecologic sonography.' [35]

###### Preservation and storage methods

'Fixation of tumor specimens followed standard protocols, using either 10% nonbuffered or 10% buffered formalin for 12 hours. Storage time of the archival samples was up to 15 years. Of the 57 independent MCL cases, 42 tumors had amplifiable cDNA.' [36]

'Tissue samples were fixed in 10% buffered formalin for 24 h, dehydrated in 70% EtOH and paraffin embedded. Five micrometer sections were cut using a cryostat (Leica Microsystems, UK) and mounted onto a histological glass slide. Ffpe [formalin-fixed, paraffin-embedded] tissue sections were stored at room temperature until further analysis.' [37]

##### Explanation

Most tumor marker prognostic studies have focused on one or more of the following types of specimens: tumor tissue (formalin fixed and paraffin-embedded or frozen); tumor cells or tumor DNA isolated from blood, bone marrow, urine or sputum; serum or plasma. Authors should report what types of specimens were used for the marker assays. As much information about the source of the specimen as possible should be included, for example, whether a tumor sample was obtained at the time of definitive surgery or from a biopsy procedure such as core needle biopsy or fine needle aspirate. For patients with advanced disease, it should be clearly stated whether tumor samples assayed came from the primary tumor site (perhaps collected years earlier at the time of an original diagnosis of early stage disease) or from a current metastatic lesion and whether the patient had been exposed to any prior cancer-directed therapies (see Item 3).

Much has been written about the potential confounding effects of pre-analytical handling of specimens, and several organizations have recently published articles addressing best practices for specimen handling [38-40]. Although the way specimens are collected is often not under the control of investigators studying prognostic markers, it is important to report as much as possible about the types of biological materials used in the study and the way these materials were collected, processed and stored. The time of specimen collection will often not coincide with the time when the marker assay is performed, as it is common for marker assays to be performed after the specimens have been stored for some period of time. It is important to state how long and how the specimens had been stored prior to performing the marker assay.

The Biospecimen Reporting for Improved Study Quality (BRISQ) guidelines provide comprehensive recommendations for what information should be reported regarding specimen characteristics and methods of specimen processing and handling when publishing research involving the use of biospecimens [41]. It is understood that reporting extensive detail is difficult if not impossible, especially when retrospective collections are used. In recognition of these difficulties, the BRISQ guidelines are presented in three tiers, according to the relative importance and feasibility of reporting certain types of biospecimen information.

Criteria for acceptability of biospecimens for use in marker studies should be established prior to initiating the study. Depending on the type of specimen and particular assay to be performed, criteria could be based on metrics such as percentage tumor cellularity, RNA integrity number, percentage viable cells or hemolysis assessment. These criteria should be reported along with a record of the percentage of specimens that met the criteria and therefore were included in the study. The numbers of specimens examined at each stage in the study should be recorded in the suggested flowchart and, particularly, in the REMARK profile (see Item 12). This information permits the reader to better assess the feasibility of collecting the required specimens and might indicate potential biases introduced by the specimen screening criteria.

Often, the specific handling of a particular set of specimens may not be known, but if the standard operating procedures of the pathology department are known, it is helpful to report information such as type of fixative used and approximate length of fixation time; both fixative and fixation time have been reported to dramatically affect the expression of some markers evaluated in tissue [42,43].

Information should be provided about whether tissue sections were cut from a block immediately prior to assaying for the marker. If tissue sections have been stored, the storage conditions (for example, temperature and air exposure) should be noted, if known. Some markers assessed by immunohistochemistry have shown significant loss of antigenicity when measured in cut sections that had been stored for various periods of time [44,45]. The use of stabilizers (for example, to protect the integrity of RNA) should be reported. For frozen specimens, it is important to report how long they were stored, at what temperature and whether they had been thawed and re-frozen. If the specimen studied is serum or plasma, information should be provided about how the specimen was collected, including anticoagulants used, the temperature at which the specimen was maintained prior to long-term storage, processing protocols, preservatives used and conditions of long-term storage.

Typically, some control samples will be assayed as part of the study. Control samples may provide information about the marker in non-diseased individuals (biological controls) or they may provide a means to monitor assay performance (assay controls).

Biological control samples may be obtained from healthy volunteers or from other patients visiting a clinic for medical care unrelated to cancer. Apparently normal tissue adjacent to the tumor tissue (in the same section) may be used or normal tissue taken during the surgical procedure but preserved in a separate block may also be used as a control. It is important to discuss the source of the biological controls and their suitability with respect to any factors that might differ between the control subjects and cancer patients (for example, other morbidities and medications, sex, age and fasting status) and have an impact on the marker [46]. Information about the comparability of handling of control samples should also be provided.

Information about assay control or calibrator samples should also be reported. For example, if dilution series are used to calibrate daily assay runs or control samples with known marker values are run with each assay batch, information about these samples should be provided (see Item 5).

### Assay methods

#### Item 5. Specify the assay method used and provide (or reference) a detailed protocol, including specific reagents or kits used, quality control procedures, reproducibility assessments, quantitation methods, and scoring and reporting protocols. Specify whether and how assays were performed blinded to the study endpoint

##### Examples

'Immunohistochemistry was used to detect the presence of p27, MLH1, and MSH2 proteins in primary tumor specimens using methods described in previous reports. Positive controls were provided by examining staining of normal colonic mucosa from each case; tumors known to lack p27, MLH1, or MSH2 were stained concurrently and served as negative controls ... In this report, we scored the tumors using a modification of our previous methods that we believe provides best reproducibility and yields the same outcome result as that using our previous scoring method (data not shown). Nuclear expression of p27 was evaluated in a total of 10 randomly selected high-power fields per tumor. A tumor cell was counted as p27 positive when its nuclear reaction was equal to or stronger than the reaction in surrounding lymphocytes, which were used as an internal control. All cases were scored as positive (>10% of tumor cells with strong nuclear staining), negative (<10% of tumor cells with strong nuclear staining), or noninformative.' [47]

'Evaluation of immunostaining was independently performed by two observers (KAH and PDG), blinded to clinical data. The agreement between the two observers was >90%. Discordant cases were reviewed with a gynaecological pathologist and were re-assigned on consensus of opinion.' [48]

##### Explanation

Assay methods should be reported in a complete and transparent fashion with a level of detail that would enable another laboratory to reproduce the measurement technique. The term 'assay' is used broadly to mean any measurement process applied to a biological specimen that yields information about that specimen. For example, the assay may involve a single biochemical measurement or multiple measurements, or it may involve a semi-quantitative and possibly subjective scoring based on pathologic assessment. It has been demonstrated for many markers that different measurement techniques can produce systematically different results. For example, different levels of human epidermal growth factor receptor 2 expression have been found using different methods [49,50]. Variations of p53 expression were observed in bladder tumors due to different staining techniques and scoring methods in a reproducibility study comparing immunohistochemical assessments performed in five different laboratories [51].

Although a complete listing of the relevant information to report for every class of assay is beyond the scope of this paper, examples of the general types of technical details that should be reported are as follows. Specific antibodies, antigen retrieval steps, standards and reference materials, scoring protocol and score reporting and interpretation (for example, if results are reported as positive or negative) should be described for immunohistochemical assays. For DNA- and RNA-based assays, specific primers and probes should be identified along with any scoring or quantitation methods used. If another widely accessible document (such as a published paper) details the exact assay method used, it is acceptable to reference that other document without repeating all the technical details. If a commercially available kit is used for the assay, it is important to state whether the kit instructions were followed exactly; any deviations from the kit's recommended procedures must be fully acknowledged in the report.

It is important to report the minimum amount of specimen that was required to perform the assay (for example, a 5 μm section or 5 μg DNA) and whether there were any other assessments that were performed to judge the suitability of the specimen for use in the study (see Item 4). Assays requiring a large amount of specimen may not be feasible for broader clinical application, and study results may be biased toward larger tumors. If there were any additional specimen pre-processing steps required (for example, microdissection or polymerase chain reaction amplification), these should be stated as well.

It is helpful to report any procedures, such as use of blinded replicate samples or control reference samples, that are employed to assess or promote consistency of assay results over time or between laboratory sites. For assays in a more advanced state of development, additional examples could include qualification criteria for new lots of antibodies or quantitative instrument calibration procedures. If reproducibility assessments have been performed, it is helpful to report the results of those studies to provide a sense of the overall variability in the assay and identify major sources contributing to the variability.

Despite complete standardization of the assay technique and quality monitoring, random variation (measurement error) in assay results may persist due to assay imprecision, variation between observers or intratumoral biological heterogeneity. For example, many immunohistochemical assays require selection of 'best' regions to score, and subjective assessments of staining intensity and percentage of stained cells. The impact of measurement error is attenuation of the estimated prognostic effect of the marker. Good prognostic performance of a marker cannot be achieved in the presence of a large amount of imprecision. It is important to report any strategies that were employed to reduce the measurement error, such as taking the average of two or three readings to produce a measurement with less error, potentially increasing the power of the study and hence the reliability of the findings. In multicenter studies, single reviewers or reference laboratories are often used to reduce variability in marker measurements, and such efforts should be noted.

There may be a risk of introducing bias when a patient's clinical outcome is known by the individual making the marker assessment, particularly when the marker evaluation involves considerable subjective judgment. Therefore, it is important to report whether marker assessments were made blinded to clinical outcome.

### Study design

#### Item 6. State the method of case selection, including whether prospective or retrospective and whether stratification or matching (for example, by stage of disease or age) was used. Specify the time period from which cases were taken, the end of the follow-up period and the median follow-up time

To clarify the discussion we have split this item into two parts.

##### a) Case selection

##### Examples

'We retrospectively analysed tumour samples from patients who were prospectively enrolled in phase II and III trials of HDC for HRPBC at the University of Colorado between 1990 and 2001.' [52]

'Seven hundred and seventy female patients with primary invasive breast cancer, diagnosed between 1992 and 1997 at the Institute of Oncology, Ljubljana, were included in the study. The patients had not been previously treated, had no proven metastatic disease at the time of diagnosis and no synchronous or metachronous occurring cancer. The primary inclusion criterion was an adequate histogram obtained from an FNA sample (see below). The diagnosis of carcinoma was therefore first established by FNA and subsequently confirmed and specified by histological examination in 690 primarily resected tumours (80 patients were not treated surgically).' [53]

'Of the 165 patients, all patients who had a pathology report of a non-well-differentiated (defined as moderately- to poorly-differentiated) SCC were identified. A matched control group of well-differentiated SCC was identified within the database. Matching criteria were (1) age (± 5 y), (2) gender, and (3) site.' [54]

##### Explanation

The reliability of a study depends importantly on the study design. An explanation of how patients were selected for inclusion in the study should be provided. Reliance on a label of 'prospective' or 'retrospective' is inadequate because these terms are ill-defined [55]. It should be clearly stated whether patients were recruited prospectively as part of a planned marker study, represent the full set or a subset of patients recruited prospectively for some other purpose such as a clinical trial or were identified retrospectively through a search of an existing database, for example from hospital or registry records or from a tumor bank. Whether patients were selected with stratification according to clinicopathologic factors such as stage, based on survival experience or according to a matched design (for example, matched pairs of patients who did and did not recur) has important implications for the analysis and interpretation, so details of the procedures used should be reported.

Authors should describe exactly how and when clinical, pathologic and follow-up data were collected for the identified patients. It should be stated whether the marker measurements were extracted retrospectively from existing records, whether assays were newly performed using stored specimens or whether assays were performed in real time using prospectively collected specimens.

In truly prospective studies, complete baseline measurements (marker or clinicopathologic factors) can be made according to a detailed protocol using standard operating procedures, and the patients can be followed for an adequate length of time to allow a comparison of survival and other outcomes in relation to baseline tumor marker values. Prospective patient identification and data collection are preferable because the data will be higher quality. Prospective studies specifically designed to address marker questions are rare, although some prognostic studies are embedded within randomized treatment trials. Aside from a potential sample size problem, a prognostic marker study may be restricted to only some of the centers from a multicenter RCT. Case selection within participating centers (for example, inclusion of only younger patients or those with large tumors) may introduce bias and details of any such selection should be reported.

Most prognostic factor studies are retrospective in the sense that the assay of interest is performed on stored samples. The benefit of these retrospective studies is that there is existing information about moderate or long-term patient follow-up. Their main disadvantage is the lower quality of the data - clinical information collected retrospectively is often incomplete and clinicopathologic data may not have been collected in a standardized fashion (except perhaps if the data were collected as part of a clinical trial). Eligible patients should be considered to be part of the study cohort and not excluded because of incomplete data or loss to follow-up, with the amount of missing data reported for each variable. That allows readers to judge the representativeness of the patients whose data were available for analysis. (See also Item 10e, Item 12, and Box 2.)

In situations where more complex case selection strategies are used, those approaches must be carefully described. Given the small size of most prognostic studies (see Item 9), it is sometimes desirable to perform stratified sampling to ensure that important subgroups (for example, different stages of disease or different age groups) are represented. The stratified sampling may be in proportion to the prevalences of the subgroups in the population, or more rare subgroups may be oversampled (weighted with a higher sampling probability), especially if subgroup analyses are planned.

Occasionally, patients are sampled in relation to their survival experience - for example, taking only patients with either very short or very long survival (excluding some patients who were censored). Simulation studies have shown that sampling which excludes certain subgroups of patients leads to bias in estimates of prognostic value and thus should be avoided [56]. If a large number of patients is available for study but few patients had events, case-control (a case being a patient with an event, a control being a patient without an event) sampling methods (matched or unmatched) may offer improved efficiency.

If standard survival analysis methods are used, unselected cases or random samples of cases from a given population are necessary to produce unbiased survival estimates. If more complex stratified, weighted or case-control sampling strategies are used, then specialized analysis methods appropriate for those sampling designs (for example, stratified and weighted analyses or conditional logistic regression) should have been applied and should be described [57,58] (see Item 10).

##### b) Time period

##### Examples

'... 1143 primary invasive breast tumors collected between 1978 and 1989 ... All patients were examined routinely every 3-6 months during the first 5 years of follow-up and once a year thereafter. The median follow-up period of patients alive (n = 584) was 124 months (range, 13-231 months). Patients with events after 120 months were censored at 120 months because after 10 years of observation, patients frequently are redirected to their general practitioner for checkups and mammography and cease to visit our outpatient breast cancer clinic.' [59]

'The estimated median follow-up time, as calculated by the reverse Kaplan-Meier method, was 4.3 years.' [60]

##### Explanation

Knowing when a study took place and over what period participants were recruited places a study in historical context. Medical and surgical therapies evolve continuously and may affect the routine care given to patients over time. In most studies where the outcome is the time to an event, follow-up of all participants is ended on a specific date. This date should be given, and it is also useful to report the median duration of follow-up.

The method of calculating the median follow up should be specified. The preferred approach is the reverse Kaplan-Meier method, which uses data from all patients in the cohort [61]. Here, the standard Kaplan-Meier method is used with the event indicator reversed so that censoring becomes the outcome of interest. Sometimes it may be helpful to also give the median follow-up of those patients who did not have the event (in other words, those with censored survival times). The amount of follow-up may vary for different endpoints, for example when recurrence is assessed locally but information about deaths comes from a central register.

It may also be useful to report how many patients were lost to follow-up for a long period (for example, over one year) or the completeness of the data compared to that if no patient was lost to follow-up [62,63].

In a review of 132 reports in oncology journals in 1991 that used survival analysis, nearly 80% included the starting and ending dates for accrual of patients, but only 24% also reported the date on which follow-up ended [64]. A review of articles published in 2006 found those dates reported in 74% and 18% of articles, respectively. Of 331 studies included in 20 published meta-analyses, the time period during which patients were selected was precisely defined in 232 (70%) [18].

#### Item 7. Precisely define all clinical endpoints examined

##### Examples

'Survival time was defined to be the period of time in months from the date of diagnosis to the date of death from breast cancer. Patients who died from causes other than those relating to breast cancer were included for the study, and data for these records were treated as right-censored cases for evaluation purposes. Relapse time was defined as the period of time in months from the date of diagnosis to the date at which relapse was clinically identified. Data on patients who dropped out of the study for reasons other than a breast-cancer relapse were considered right-censored for these analyses.' [65]

'The primary end point was tumour recurrence or death of a patient. RFS was defined as time from mastectomy to the first occurrence of either locoregional or distant recurrence, contralateral tumour, secondary tumour or death; overall survival as time from operation to death.' [66]

##### Explanation

Survival analysis is based on the elapsed time from a relevant time origin, often the date of diagnosis, surgery or randomization, to a clinical endpoint. That time origin should always be specified.

Most prognostic studies in cancer examine few endpoints, mainly death, recurrence of disease or both, but these end-points are often not clearly defined (see Box 3). Analyses of time to death may be based on either deaths from any cause or only cancer related deaths. The endpoint should be defined precisely and not referred to just as 'survival' or 'overall survival'. If deaths from cancer are analyzed, it is important to indicate how the cause of death was classified. If known, it can also be helpful to indicate what records (such as death certificate or tumor registry) were examined to determine the cause of death.

If there was a specific rationale for choosing the primary clinical endpoint, it should be stated. For example, if the studied marker is believed to be associated with the ability of a cell to metastasize, an endpoint that focuses on distant recurrences might be justified. For a marker believed to be associated with sensitivity to radiation therapy, local-regional recurrences in a population of patients who received radiotherapy following primary surgery might be relevant.

The lack of standardized definitions also affects the analysis of recurrence of disease. Relapse-free survival, disease-free survival (DFS), remission duration and progression-free survival are the terms most commonly used; however, they are rarely defined precisely. The first three imply that only patients who were disease-free after initial intervention were analyzed (although this is not always the case), while for progression-free survival all patients are generally included in the analysis. If authors analyze disease recurrence they should precisely define that endpoint, in particular with respect to how deaths are treated. Similarly, outcomes such as distant DFS should be defined precisely. Further, standardized definitions across studies would be desirable [67].

Some endpoints require subjective determination (for example, progression-free survival determined by a review of radiographic images). For this reason, it can also be helpful to report, if known, whether the endpoint assessments were made blinded to the marker measurements. It is helpful to report any additional steps taken to confirm the endpoint assessments (for example, a central review of images for progression determination).

The time origin was not stated for at least one endpoint in 48% of 132 papers in cancer journals reporting survival analyses [64]. At least one endpoint was not clearly defined in 62% of papers. Among the 106 papers with death as an endpoint, only 50 (47%) explicitly described the endpoint as either any death or only cancer death. In 64 papers that reported time to disease progression, the treatment of deaths was unclear in 39 (61%). Outcomes were precisely defined in 254 of 331 studies (77%) included in 20 published meta-analyses [18]. The authors noted, however, that 'this percentage may be spuriously high because we considered all mortality definitions to be appropriate regardless of whether any level of detail was provided'.

#### Item 8. List all candidate variables initially examined or considered for inclusion in models

##### Example

'Cox survival analyses were performed to examine prognostic effects of vitamin D univariately (our primary analysis) and after adjustment for each of the following in turn: age (in years), tumor stage (T2, T3, or TX v T1), nodal stage (positive v negative), estrogen receptor status (positive or equivocal v negative), grade (3 v 1 or 2), use of adjuvant chemotherapy (any v none), use of adjuvant hormone therapy (any v none), body mass index (BMI; in kilograms per square meter), insulin (in picomoles per liter), and season of blood draw (summer v winter). Simultaneous adjustment for age, tumor stage, nodal stage, estrogen receptor status, and grade was then performed.' [68]

##### Explanation

It is important for readers to know which marker measurements or other clinical or pathological variables were initially considered for inclusion in models, including variables not ultimately used. The reasons for lack of inclusion of variables should be addressed; for example, variables with large amounts of missing data (see Box 2). Authors should fully define all variables and, when relevant, they should explain how they were measured.

All of the variables considered for standard survival analyses should be measured at or before the study time origin (for example, the date of diagnosis) [69,70]. (For tumor markers, this means the measurements are made on specimens collected at or before study time origin even if the actual marker assays are performed at a later time on stored specimens.) Variables measured after the time origin, such as experiencing an adverse event, should more properly be considered as outcomes, not predictors [71]. Another example is tumor shrinkage when the time origin is diagnosis or start of treatment. Statistical methods exist to allow inclusion of variables measured at times after the start of follow-up ('time-dependent covariates') [72], but they are rarely used and require strong assumptions [73,74].

A list of the considered candidate variables was presented in 71% of a collection of 331 prognostic studies [18]. Of 132 articles published in cancer journals, 18 (13%) analyzed variables that were not measurable at the study time origin [64], of which 15 compared the survival of patients who responded to treatment to survival of those who did not respond. Out of 682 observational studies in clinical journals that used a survival analysis, 127 (19%) included covariates not measurable at baseline [69].

#### Item 9. Give rationale for sample size; if the study was designed to detect a specified effect size, give the target power and effect size

##### Examples

'Cost and practical issues restricted the sample size in our study to 400 patients. Only 30 centres entered ten or more patients in AXIS, so for practical reasons, retrieval of samples began with these centres within the UK, continuing until the target sample size of 400 had been reached.' [75]

'Assuming a control survival rate of 60% and 50% of patients with high TS expression or p53 overexpression, then analysis of tissue samples from 750 patients will have 80% power to detect an absolute difference of 10% in OS associated with the expression of either of these markers.' [76]

'Although it was a large trial, FOCUS still lacked power to be split into test and validation data sets. It was therefore treated as a single test-set, and positive findings from this analysis need to be validated in an independent patient population. A 1% significance level was used to allow for multiple testing. The number of assessable patients, variant allele frequencies, and consequent power varied by polymorphism; however, with an overall primary outcome event rate of 20%, we could detect differences of 10% (eg, 14% *v *24%) between any two treatment comparisons, and we could detect a linear trend in genotype subgroups varying by 6% (eg, 13% *v *19% *v *25%) with a significance level of 1% and 90% power ... Even with a dropout rate of 14% for incomplete clinical data, there was 85% power at a significance level of 1% to detect a 10% difference from 14% to 24% in toxicity for any two treatment comparisons or a linear trend in genotype subgroups from 13% to 19% to 25%.' [77]

##### Explanation

Sample size has generally received little attention in prognostic studies, perhaps because these studies are often performed using pre-existing specimen collections or data sets. For several reasons, the basis for a sample size calculation in these studies is less clear than for a randomized trial. For example, the minimum effect size of interest for a prognostic marker study may be quite different from that of an intervention study, and the effect of the marker adjusted for other standard variables in a multivariable model may be of greater interest than the unadjusted effect. Authors should explain the considerations that led to the sample size. Sometimes a formal statistical calculation will have been performed, for example calculation of the number of cases required to obtain an estimated hazard ratio with prescribed precision or to have adequate power to detect an effect of a given size. More often sample size will be determined by practical considerations, such as the availability of tumor samples or cost. Even in this situation, it is still helpful to report what effect size will be detectable with sufficient power given the pre-determined sample size.

Several authors have addressed the issue of sample size calculations applicable to prognostic studies [78-80]. The most important factor influencing power and sample size requirement for a study with a time-to-event outcome is the number of observed events (effective sample size), not the number of patients. For a binary outcome, the effective sample size is the smaller of the two frequencies, 'event' or 'non-event'. Additional factors, such as the minimum detectable effect size, distribution of the marker (or the prevalence of a binary marker), coding of the marker (whether treated as a continuous variable or dichotomous; see Item 11 and Box 4) and type of analysis method or statistical test also have an impact. As a consequence of the importance of the number of events, studies of patients with a relatively good prognosis, such as lymph node negative breast cancer, require many more patients or longer follow-up than studies of metastatic disease in which events are more frequently observed. Choice of an endpoint that includes recurrence as an event in addition to death will also result in more observed events and higher power, an important reason as to why DFS is often preferred as an endpoint [81].

Sample size requirements will differ depending on the goal of the study and stage of development of the marker. For markers early in the development process, investigators may be most interested in detecting large effects unadjusted for other variables and may be willing to accept higher chances of false positive findings (that is, a higher type I error) to avoid missing interesting marker effects. Targeting larger effect sizes and allowing higher error rates will result in a smaller required sample size. As a prognostic marker advances in the development process, it will typically be studied in the context of regression models containing other clinically relevant variables, as discussed in Item 10d. These situations will require larger sample sizes to account for the diminished size of marker effects adjusted for other (potentially correlated) variables and to offer some stability even when multiple variables will be examined and model selection methods will be used.

When the goal is to identify the most relevant variables in a model, various authors have suggested that at least 10 to 25 events are required for each of the potential prognostic variables to be investigated [82-85]. Sometimes the primary focus is estimation of the marker effect after adjustment for a set of standard variables, so correctly identifying which of the other variables are really important contributors to the model is of less concern. In this situation, sample size need not be as large as the 10 to 25 events per variable rule would recommend [86] and other sample size calculation methods that appropriately account for correlation of the marker with the other variables are available [78,87]. Required sample sizes are substantially larger if interactions are investigated. For example, an interaction between a marker and a treatment indicator may be examined to assess whether a marker is predictive for treatment benefit (see Box 3).

Several studies have noted the generally small sample size of published studies of prognostic markers. In a review of lung cancer prognostic marker studies, the median number of patients per study was 120 [88], while three quarters of studies in a review of osteosarcoma prognostic marker literature included fewer than 100 patients [89]. In a systematic review of tumor markers for neuroblastoma, 122 (38%) of 318 eligible reports were excluded because the sample size was 25 or lower [90]. As mentioned above, the number of events is a more relevant determinant of power of a study, and it is usually much smaller and often not even reported (see Item 12).

Twenty meta-analyses that included 331 cancer prognostic studies published between 1987 and 2005 were assessed to determine the quality of reporting for the included studies [18]. Only three (0.9%) of the 331 studies reported that a power calculation had been performed to determine sample size.

### Statistical analysis methods

#### Item 10. Specify all statistical methods, including details of any variable selection procedures and other model-building issues, how model assumptions were verified, and how missing data were handled

After some broad introductory observations about statistical analyses, we consider this key item under eight subheadings.

All the statistical methods used in the analysis should be reported. A sound general principle is to 'describe statistical methods with enough detail to enable a knowledgeable reader with access to the original data to verify the reported results' [91]. It is additionally valuable if the reader can also understand the reasons for the approaches taken.

Moreover, for prognostic marker studies there are many possible analysis strategies and choices are made at each step of the analysis. If many different analyses are performed, and only those with the best results are reported; this can lead to very misleading inferences. Therefore, it is essential also to give a broad, comprehensive view of the range of analyses that have been undertaken in the study (see also the REMARK profile in Item 12). Details can be given in supplementary material if necessary due to publication length limitations.

Analysis of a marker's prognostic value is usually more complex than the analysis of a randomized trial, for which statistical principles and methods are well developed and primary analysis plans are generally pre-specified. Many of the marker analysis decisions can sensibly be made only after some preliminary examination of the data and therefore generally only some key features of the analysis plan can be pre-specified. Many decisions will be required, including coding of variables, handling of missing data and specification of models. It would be useful to clarify which of these decisions were pre-specified and which were made *post hoc *or even in deviation from the original analysis plan.

Reporting of key features of an analysis is important to allow readers to understand the reasons for the specific approach chosen and to assess the results. No study seems yet to have investigated in detail the large variety of statistical methods used and the quality of their reporting, but the common weaknesses in applying methods and the general insufficient reporting of statistical aspects of a multivariable analysis have been well known for many years. Empirical investigations of published research articles seem to concentrate more on randomized trials and epidemiological studies, but the methods and problems of multivariable models in the latter are similar to prognostic studies. Concato *et al*. identified 44 articles which considered risk factors in the framework of a logistic regression model or a proportional hazard model [92]. All had at least one severe weakness, and they concluded 'the findings suggest a need for improvement in the reporting and perhaps conducting of multivariable analyses in medical research'. Recently Mallett and colleagues assessed 50 articles reporting tumor marker prognostic studies for their adherence to some items from the REMARK checklist [20]. In 49 out of 50 studies (98%), the Cox model was used. Proportional hazards is one of the key assumptions of this model but only four articles (8%) reported testing this assumption (see Item 18). Sigounas *et al*. assessed 184 studies on prognostic markers for acute pancreatitis. Multivariable analyses were performed in only 15 of them, of which only one provided all details requested in Item 10 [21]. Although bad reporting does not mean that bad methods were used, the many studies identifying specific issues of bad reporting clearly show that a substantial improvement of reporting of statistical methods is needed [18,21,33,64,93-98].

In the following sections we consider specific aspects of analyses under eight headings. Not all aspects will be relevant for some studies. More extensive discussions of statistical analysis methods for binary outcome and for survival data can be found elsewhere [73,99-111].

##### a. Preliminary data preparation

##### Example

'Ki67 was measured as a continuous score which is typically positively skewed. Analysis was undertaken by log transforming Ki67 and using log(Ki67) as a covariate to investigate whether there is a linear increase in the probability of relapse with increasing Ki67 value.' [112]

##### Explanation

Some assessment of the data quality usually takes place prior to the main statistical analyses of the data, and some data values may be changed or removed if they are deemed unreliable. These manipulations and pre-modeling decisions could have a substantial impact on the results and should be reported, but rarely are [113-117].

There are many examples of steps typically taken in initial data analyses. The distribution of the marker values and distributions of any other variables that will be considered in models should be examined for evidence of extreme values or severe skewness. It may be appropriate to truncate or omit extreme outliers. Preliminary transformations of specific variables (for example, logarithm or square root) may be applied to remove severe skewness. For categorical variables, re-categorization is often performed to eliminate sparse categories (for example, histological types of tumors). Graphical representations or summary statistics calculated to assess the distribution of the marker or other variables (for example, boxplot; mean, median, SD, range and frequencies) should be described because different methods will depict features of the data with varying degrees of sensitivity (such as outliers and skewness). If some marker measurements were judged to be unreliable and consequently omitted or adjusted to lessen their influence in the analysis, it is recommended these details be reported as they can be informative about the robustness of the assay and stability of the analysis results. It is helpful to report these early steps of the analysis along with the number of data values that were excluded or somehow modified (see also Items 12 and 13).

##### b. Association of marker values with other variables

##### Example

'The associations of cathepsin-D with other variables were tested with non-parametric tests: with Spearman rank correlation (r_s_) for continuous variables (age, ER, PgR), and the Wilcoxon rank-sum test or Kruskal-Wallis test, including a Wilcoxon-type test for trend across ordered groups where appropriate, for categorical variables.' [29]

##### Explanation

Early steps in an analysis may include an examination of the relationship of the marker to other variables being considered in the study. These variables might include established clinical, pathologic, and demographic covariates (see Items 13 and 14). If more than one marker is being evaluated in a study, the relationships between the multiple markers should be examined.

Methods for summarizing associations with other variables (for example, correlation coefficients, chi-square tests and t-tests) should be described. Extreme or unusual associations may be relevant to the validity of analyses and stability of results and may suggest further data modifications are advisable (see section a above) or that certain variables are redundant.

##### c. Methods to evaluate a marker's univariable association with clinical outcome

##### Example

'Median survival time and median DFI [disease free interval] for the whole test set were estimated using the Kaplan-Meier product limit method. Univariate associations between survival time, DFI, and glucose were examined using Cox proportional hazards regression models. These analyses examined glucose as a continuous variable, using an increment of 70 mg/dL to derive hazard ratios, and adjusted for time of blood draw to control for circadian effects on glucose levels ... Wald Chi-square P values were used to calculate univariate statistical significance, and 95% confidence intervals were estimated.' [118]

##### Explanation

A marker's association with clinical outcome is of key importance. The first evaluation will usually be conducted without adjustment for additional variables, that is, a univariable analysis. The method of analysis (for example, logrank test or estimated effect with confidence interval in a Cox regression or a parametric model for survival data), including options such as choice of test statistic (for example, Wald test, likelihood ratio test or score test), should be reported.

Any variable codings or groupings, or transformations of continuous values applied to the marker variable or any other variables, should be stated to allow for proper interpretation of the estimated associations (see Box 4 and Item 11).

In addition, similar analyses may be conducted to examine the association of other variables with clinical outcome.

##### d. Multivariable analyses

##### Examples

'A Cox regression model was used with individual marker as the exposure variables and OS [overall survival] (from time of surgery to time of death or end of current follow-up) as the outcome. The analyses were adjusted simultaneously for sex, age, tumour size, grade (World Health Organization), stage and sites as well as use of post-operative adjuvant therapies.' [76]

'Univariable and multivariable Cox regression models addressed CSM after NU or SU. Covariates consisted of pathologically determined T stage (pT1 versus pT2 versus pT3 versus pT4), N stage (N0 versus N1-3), tumour grade (I versus II versus III versus IV), primary tumour location (ureter versus renal pelvis), type of surgery (NU with bladder cuff versus NU without bladder cuff versus SU), year of surgery, gender (male versus female) and age. Since pT and pN stages, as well as tumour grade, may contribute to a multiplicative increase in CSM rate, we tested three first-degree interactions between these variables. Specifically, multivariable interaction tests were performed between pT and pN stages, between T stage and tumour grade and between N stage and tumour grade.' [119]

'For both models 1 and 2 a competing risk analysis was performed using cause-specific hazards. This analysis follows separate Cox models for each event assuming proportional hazards. In such competing risks analyses with two endpoints, it is possible to interpret both cause-specific hazard ratios simultaneously for each risk factor. Cumulative incidence functions have been displayed for each endpoint. The proportional hazard assumptions were assessed by study of the graphs of the Schoenfeld's residuals; this technique is especially suitable for time-dependent covariates.' [120]

##### Explanation

Univariable analyses are useful but, except in early studies, are generally insufficient because of the possible relationship of the marker with other variables. Thus the prognostic value of the marker after adjustment for established prognostic factors, as estimated from a multivariable model (see Item 17), will be of major interest. To facilitate comparison of the unadjusted and adjusted measures of association, it is helpful to report results from univariable analyses that used the same general approach as the approach used for the multivariable analysis. For example, if multivariable analyses adjusting for standard prognostic factors are based on a Cox regression model with the log-transformed marker value as one of the independent variables, then it is helpful also to report the corresponding results of a univariable Cox regression analysis. This allows for direct assessment of how the marker's regression coefficient is altered by inclusion of standard covariates in the model.

Whereas the Cox proportional hazards model allows a flexible form of baseline hazard, parametric models assume specific functional forms [109,121,122]. Parametric models [123] will be statistically more efficient if the model is correct and may be more easily adaptable to situations involving complex censoring patterns, but if the assumed functional form of the baseline hazard is incorrect, they can be misleading. It is important that authors report which model was used.

Multivariable methods can also be used to build prognostic models involving combinations of several candidate markers or even many hundreds of markers (for example, gene expression microarray data). Although the same basic analysis principles apply to these situations, even greater care must be taken to ensure proper fit of such models and avoid overfitting, and to rigorously evaluate the model's prognostic performance. These topics are covered in many articles and books [99,101,108,110,124-126] and are not a focus of this paper.

Investigators may use statistical approaches other than classic multivariable regression to take into account multiple variables. Such techniques include classification and regression trees and artificial neural networks. Their detailed discussion is beyond the scope of the current guidelines; for details the reader is referred elsewhere [107].

##### e. Missing data

##### Example

'Thirteen patients (all either ductal carcinoma, lobular carcinoma or mixed histology) had no grade information recorded in the data and one patient had no tumour size recorded. These patients were included in the analysis using multiple imputation methods to estimate the missing values. The hazard ratios were derived from the average effect across 10 augmented datasets, with the confidence intervals and significance tests taking into account the uncertainty of the imputations. The multiple imputation was performed by the MICE library within the S-Plus 2000 Guide to Statistics Volumes 1 and 2 (MathSoft, Seattle, WA, USA) ... ' [127]

##### Explanation

Almost all prognostic studies have missing marker or covariate data for some patients because clinical databases are often incomplete. Also, some marker assays may not yield interpretable results for all specimens. However, not all papers report in detail the amount of missing data and very few attempt to address the problem statistically [33].

Authors should report the number of missing values for each variable of interest. They should give reasons for missing values if possible, and indicate how many individuals were excluded because of missing data when describing the flow of participants through the study (see Item 12). Many authors omit cases without all relevant information from all analyses or they may vary who is included according to which variables are included in the analysis. Including only cases with complete data may greatly reduce the sample size and potentially lead to biased results if the likelihood of being missing is related to the true value (see Box 2) [33,128-131]. Modern statistical methods exist to allow estimation (imputation) of missing observations. These issues are clarified in Box 2. Authors should describe the nature of any such analysis (for example, multiple imputation) and specify assumptions that were made (for example, 'missing at random').

In a review of 100 prognostic articles, the percentage of eligible cases with complete data was obtainable in only 39; in 17 of these articles more than 10% of patients had some missing data. The methods used to handle incomplete covariates were reported in only 32 out of 81 articles with known missing data [33].

##### f. Variable selection

##### Example

'When using a stepwise variable selection procedure to identify independent factors prognostic for survival, variables were added using forward selection according to a selection entry criterion of 0.05 and removed using backward elimination according to a selection stay criterion of 0.05. The importance of a prognostic factor was assessed via Wald-type test statistics, the hazard ratio and its 95% confidence interval for survival.' [132]

##### Explanation

Sometimes several multivariable models containing different subsets of variables are considered. The rationale for these choices and details of any model selection strategies used should be described. The REMARK profile can provide a concise summary of all analyses performed (Item 12).

If patients in the study received different treatments, one or more variables indicating treatments received can be considered in models, treatment can be used as a stratification factor or separate models may be built for each treatment. For many cancer types, there are a few generally accepted staging variables or other clinical or pathologic variables that would be available in most cases, and these variables would usually be considered in multivariable models (see also Item 17).

The main multivariable model may sometimes be pre-specified, which helps to avoid biases caused by data-dependent model selection. More often, however, many candidate variables are available and some type of variable selection procedure is sensible in order to derive simpler models which are easier to interpret and may be more generally useful [108,133]. It is particularly important to state if the variables included in a reported model were determined using variable selection procedures. Any selection procedures used should be described (for example, stepwise regression or backward elimination) along with specific criteria used to determine inclusion or exclusion of variables from the model (for example, *P *values) or to select a best fitting model (for example, Akaike information criterion) [101]. It is well known that, unless sample sizes are large, use of variable selection procedures will lead to biased parameter estimates and exaggerated measures of statistical significance [66,121,134]. For this reason, Item 17 requests that results from a particular multivariable model which includes the marker along with 'standard' prognostic variables, regardless of statistical significance, be reported.

##### g. Checking model assumptions

##### Examples

'In the basic form of the Cox regression model, the coefficients corresponded to the logarithm of the HR and were constant in time. This assumption was graphically evaluated by means of smoothed Schoenfeld residuals and tested as suggested by Grambsch and Therneau.' [135]

'The proportional hazards assumptions were checked by plots of log(- log survival time) versus log time.' [136]

'We evaluated the proportional hazards assumption by adding interaction terms between the time-dependent logarithm of follow-up time plus 1 and tamoxifen treatment, ERαS118-P status, or both and found no evidence for nonproportional hazards (P = .816, .490, and .403, respectively).' [24]

##### Explanation

Any statistical model, univariable or multivariable, makes certain assumptions about the distributions of variables or the functional relationships between variables. For example, the Cox proportional hazards regression model commonly used for survival data requires several important assumptions, including proportional hazards and linear relationships between continuous covariates and the log hazard function. Proportional hazards assumptions are often violated when there is long follow-up, for example, for certain types of cancers in which a portion of patients can be considered cured. How the variables are coded or transformed will also affect the appropriateness of linear versus non-linear relationships (see Item 11 and Box 4).

Methods used to empirically check model assumptions should be reported. For example, residual plots and models containing time-by-covariate interactions are often used to diagnose departures from linearity and proportional hazards [122,137-139]. Influential points and outliers can often be detected by diagnostic plots such as added variable plots [140]. Parametric survival models, such as lognormal or Weibull models, make additional assumptions about the distribution of the survival times [123]. The suitability of parametric models can be checked using methods such as residual plots and goodness of fit tests [109,121]. Many extensions of the Cox model have been proposed to handle departures from the basic assumptions [138,139] but they will not be discussed here. More complex models require larger sample sizes than often are available in tumor marker prognostic studies to avoid overfitting to noise in the data [107,141].

Alternative models evaluated for purposes of sensitivity analyses should also be described (see Item 18).

##### h. Model validation

##### Examples

'For internal validation of the multivariate models, 1000 bootstrap samples were created and stepwise Cox regression analysis was applied to each sample. The relative frequencies of inclusions of the respective factors were calculated.' [142]

'For this study, and future studies using this TMA, the primary investigator is given access to all clinical, outcome, and TMA data from the training set only. The training set is used to generate and refine hypotheses regarding the biomarker under study. Significant findings are then formally presented ... Those findings considered to be of clinical and scientific interest are then re-tested on the validation set. A separate researcher who did not participate in the training set analysis performs the re-testing on the validation set. Our statistical approach is intended to minimize false positive results, particularly with subgroup analysis.' [143]

##### Explanation

Invariably, the strongest evidence for the validity of results is confirmation of the findings on data not involved in the original analysis [144,145]. The ideal approach is to confirm findings from the main (final) model on completely independent data, preferably collected by different investigators but under pre-defined appropriate conditions. If successful, this approach would indicate that the results are transportable to other settings. This would be a type of 'external validation'. A prospectively designed and conducted clinical trial is the strongest form of validation, but trials designed with the primary objective to validate a prognostic marker or model are rare. More often, evaluations of markers occurring within trials are secondary aims in trials primarily designed to evaluate a treatment or other intervention. The marker evaluation could occur during the trial, or the evaluation might take place even years after completion of the trial using specimens banked during the course of the trial. This latter option has been referred to as a 'prospective-retrospective' design, and it can provide a high level of evidence for the utility of a marker if conducted under appropriate conditions [146]. Complete specification of the marker assay method and model (if relevant), a pre-specified analysis plan, and enforcement and documentation of lock-down of marker analytical results prior to unblinding of clinical outcome data (see also Item 5) are among the conditions that should be satisfied for a rigorous prospective-retrospective validation.

A completely independent data set (a 'similar' study) often will not be available, but 'internal' validation procedures, such as cross-validation, bootstrapping or other data resampling methods [133,147], are useful to give insights into critical issues such as bias of regression parameter estimates, overoptimism of prognostic model discriminatory ability or stability of the model derived (see also Item 18). Internal validation involves holding out some portion of the data ('test set') while a model is built on the remaining portion ('training set'); when the model is completely specified on the training set, it is then evaluated (tested) on the held-out data. A limitation of internal validation is that there may be biases affecting the entire data set that will not be detected by internal validation because the biases will affect the training and test sets equally [46]; however, if a model has been seriously overfitted to random noise in the training set, properly performed internal validation should reveal failure of the model on the test data. The study report should include a description of any validations that were performed, internal or external.

For internal validation, the specific validation algorithm used should be described (for example, bootstrapping, 10-fold or leave-one-out cross-validation) [147-149]. If a study performs any external validation, basic details of the study population, design and analysis approach should be provided. It should be clarified whether the external validation sample came from the same or different centers or periods as the samples used to develop the model. In cases where the whole study represents a validation of a previously developed model this should be stated, along with proper reference to the previous study that developed that model.

#### Item 11. Clarify how marker values were handled in the analyses; if relevant, describe methods used for cutpoint determination

##### Examples

'In the regression models, steroid receptors content and age were considered as continuous variables, the latter in its original measure scale and the former in terms of its natural logarithms because of the positive skew of its distribution. Null values for steroid receptor content were arbitrarily set to 1 considering a sensitivity threshold value of 2 fmol/mg of cytosolic protein.' [135]

'Hazard ratios (HRs) and 95% CIs for CRP and SAA tertiles were estimated using Cox proportional hazards regression ... CRP and SAA values were log transformed to account for skewness, and HRs and 95% CIs were generated for these continuous measures.' [150]

'As there was no clinically defined cutoff point for serum IL-6 level, the median was used to divide the patients into two groups (low versus high serum IL-6 level).' [151]

'In the absence of a reliable gold standard and following distributional studies, we used the 25th percentile of observed hormonal receptor mRNA expression levels and the median of observed MAP-Tau mRNA levels as thresholds for categorization of tumors to positive or negative cases.' [152]

##### Explanation

Many markers are measured as continuous variables. A central question is how to analyze these variables, including how to incorporate them in a multivariable model. The same considerations apply to several standard variables, such as age and tumor size.

Two main approaches are to keep the variables as continuous (but not necessarily assume a linear relation with the outcome), or to group the data into categories. Although categorization is ubiquitous in cancer studies, there are some major concerns about that approach, as discussed in Box 4 [153]. The common practice of using only two categories makes it impossible to detect any nonlinearity in the relation between the variable and outcome. However, for later clinical use, dichotomization may be necessary.

Authors should report how each continuous variable was incorporated into the analyses. For categorized variables, they should specify the cutpoints and how they were chosen. It is especially important to declare any cutpoints chosen after examining many options (see Box 4). For continuous variables, authors should clarify whether the data were kept on the original scale or, say, log transformed, and indicate whether the relationship was modeled as linear or non-linear, and how. If treated as linear, it is helpful to report whether the assumption of a linear relationship for continuous variables was checked (Box 4).

Similar concerns relate to variables with three or more ordered categories, such as Karnofsky score. For markers and other variables with several categories (for example, from three to six) it is important to specify how they were treated in the analyses. If dummy variables were created, it is important to specify how they were defined and analyzed [110]. If multiple methods of coding dummy variables are considered in the analysis, there is a risk of selective reporting of the results that look most interesting.

Reviews of published prognostic factor studies show that categorization is very common, with almost all studies reporting results for dichotomized marker values [15]. Further, there is usually considerable variation in cut-off values across studies, hindering a sound comparison of results. For example, a review of p53 in bladder cancer found that definitions of positive p53 staining cut-off values ranged from 1% to 75% [154].

## Results

### Data

#### Item 12. Describe the flow of patients through the study, including the number of patients included in each stage of the analysis (a diagram may be helpful) and reasons for dropout. Specifically, both overall and for each subgroup extensively examined report the number of patients and the number of events

##### Examples

'Tumor samples from 375 patients were sent to the central laboratory for EGFR assays by IHC, and evaluable assay results were obtained for 325 patients (87%). Among the 50 patients with unevaluable results, 38 (76%) had insufficient tumor cells in their tumor sample, six (12%) had extensive necrosis, three (6%) had inadequate control staining, two (4%) had poor tumor preservation, and one (2%) had a broken slide.' [155]

See also Figure 1.

**Figure 1:**
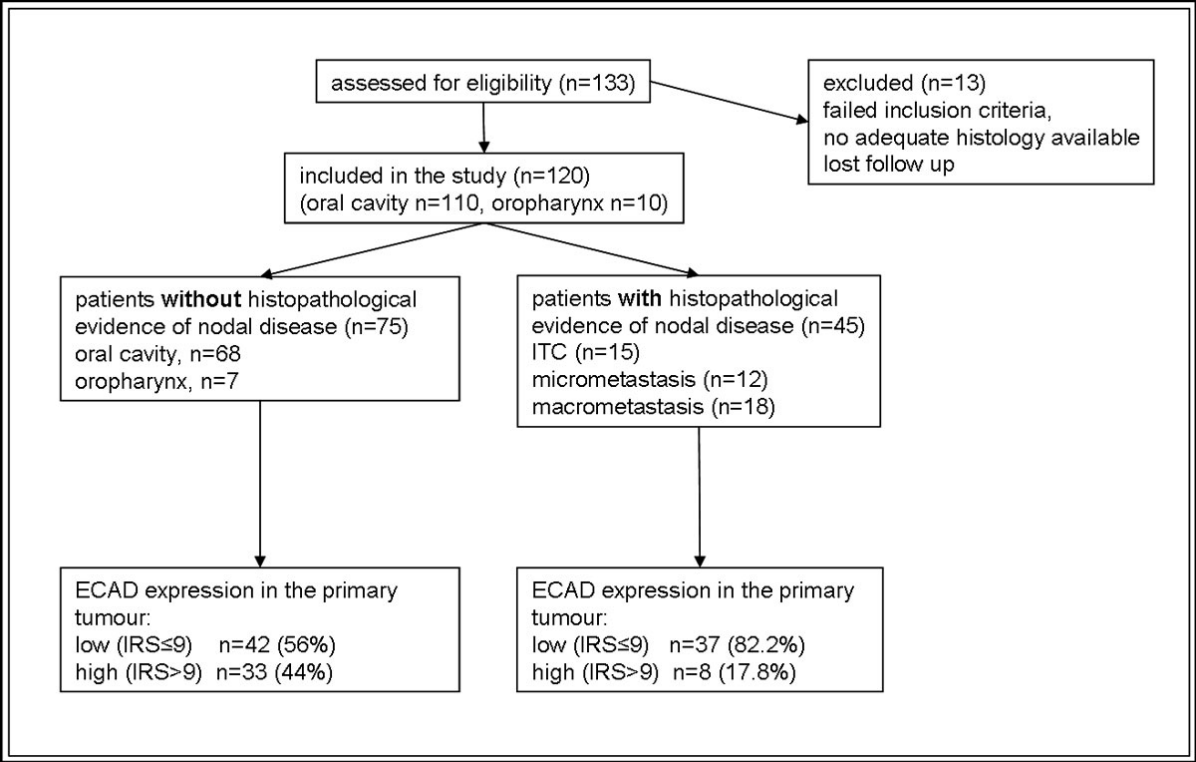
**Example of a participant flow diagram**. [177]

##### Explanation

The interpretation of prognostic studies depends on having a good understanding of the patients included in the study, the methods used, the analyses conducted and the amount of data available at each stage. In contrast to RCTs, exploratory analyses play a much more important role (see Item 10). In general, several analyses are conducted of which only some are fully reported, with the results of others mentioned only briefly in the text (and easily overlooked) or not reported at all. This selective reporting practice gives rise to biased results and biased interpretation and should be avoided. Important information, such as the effective sample size (see Item 9), is usually not given for many analyses. At present, hardly any report fully meets the needs of readers [20,21].

One way to ensure completeness of reporting of key information is via a structured display. Even for RCTs, which are relatively straightforward, it is often impossible to understand from the text why the numbers of patients in analyses differs from the numbers enrolled in the trial. Thus the CONSORT flow diagram [8] has become a widely used simple depiction of the flow of participants in an RCT from enrolment through to inclusion in the final analysis.

Analyzing and reporting prognostic studies is in general more complicated than for RCTs. Therefore, we suggest two complementary displays that authors can use to summarize key aspects of a prognostic study, especially the derivation of the sample and details of the analyses performed. A flow diagram provides an easy to follow view of the major changes in the population as the study proceeds; a study profile (see below) provides a succinct summary of the analyses performed and the data used in them.

The upper part of a study profile can be used to show the derivation of the sample of patients included in the study. It is analogous to the CONSORT [8] and STROBE [156] flow diagrams, but gives the information in a more condensed way and may make a flow diagram, as shown in the examples, redundant (see also Item 2). Its inclusion in reports of prognostic studies would help to clarify the extent to which the analyzed patients were selected from a larger series.

Knowing how many patients were included in a study is important, but information should be given about the amount of data available for each analysis. Missing values (see Box 2) are much more common in retrospective studies than in prospective studies due to the use of historical data. The complete case analysis is the most widely used method (see Item 10e); as a consequence, the number of patients and events will often vary across analyses according to the choice of adjusting variables. Further, the outcome measure and any restriction to a subgroup also affect the number of patients and events. These numbers are a key element determining the statistical reliability of any analysis. Readers thus need to understand which patients (and how many events) were included in each analysis, and also which variables were used. For all of these reasons, a standard format for reporting all analyses performed would be extremely helpful and is strongly recommended.

We developed a two-part study profile which has already been used in a paper on the reporting of prognostic studies [20]. As illustrated in the examples below, the first part gives details about how the marker of interest was handled in the analysis and which further variables were available. In addition, key information can usefully be provided in this part about the patient population, inclusion and exclusion criteria, and the number of eligible patients and events for each outcome in the full data set. In the first example (Table 2), the number and reasons for patients excluded are given, but not the numbers for each reason. These numbers can easily be given and would help readers to assess a study.

**Table 2:** Example of the REMARK profile illustrated using data from a study of ploidy in patients with advanced ovarian cancer [157] (from [20]).

a) Patients, treatment and variables				
Study and marker	Remarks			
Marker (If non-binary: how was marker analyzed? continuous or categorical. If categorical, how were cutpoints determined?)	M = ploidy (diploid, aneuploid)			
Further variables (variables collected, variables available for analysis, baseline variables, patient and tumor variables)	v1 = age, v2 = histologic type, v3 = grade, v4 = residual tumor, v5 = stage, v6 = ascites^a^, v7 = estrogen^a^, v8 = progesterone^a^, v9 = CA-125^a^			

**Patients**	**n**	**Remarks**		

Assessed for eligibility	257	*Disease*: Advanced ovarian cancer, stage III and IV *Patient source*: Surgery 1982 to 1990, University Hospital Freiburg *Sample source*: Archived specimens available		
Excluded	73	General exclusion criteria^b^, non-standard therapy^b^, coefficient of variation > 7%^b^		
Included	184	Previously untreated. *Treatment*: all had platinum based chemotherapy after surgery		
With outcome events	139	Overall survival: death from any cause		

**b) Statistical analyses of survival outcomes**				
**Analysis**	**Patients**	**Events**	**Variables considered**	**Results/remarks**

A1: Univariable	184	139	M, v1 to v5	Table 2, Figure 1
A2: Multivariable	174	133	M, v1, v3 to v5	Table 3 [v2 omitted because many missing data; Backward selection, see text]
A3: Effect for ploidy adjusted for v4	184	139	M, v4	Figure 2 [Based on result of A2]
A4: Interaction: ploidy and stage	175	133	M, v1, v2, v4, v5	See text
A5: Ploidy in stage subgroups				
v5 = III	128	88	M	Figure 3
v5 = IV	56	51	M	Figure 4

^a^Not considered for survival outcome as these factors are not considered as 'standard' factors and/or number of missing values was relatively large; ^b^values not given in the paper.

As the patient population is often heterogeneous with regard to stage of the disease, treatment and other factors, it is common practice to assess the marker in several more homogeneous subgroups of the population. Furthermore, several outcomes (for example, DFS, distant DFS or overall survival, OS) are usually considered. Figures showing Kaplan-Meier estimates are often presented for a univariable assessment, for a continuous marker divided into subgroups. However, the results of further analyses and details about variables in a multivariable model are often only briefly summarized in the text or perhaps not mentioned at all. (See Box 5 for discussion of the implications of selective reporting.)

To help the reader understand the multiplicity of analyses and better assess the results, the second part of the proposed profile gives an overview of all analyses. Nearly all reports of prognostic marker studies include univariable, multivariable and subgroup analyses. Several multivariable analyses are often reported in prognostic marker studies. It is critical to know which variables were available in order to determine the most appropriate multivariable analysis for a given study. Also, it is frequently unclear which variables have been adjusted for in each analysis. Often, some analyses and their results are mentioned in just one sentence in the text (for example, 'the effect of marker x was the same in subgroup A' or 'the effect of marker x was unchanged when adjusting for the three variables v1, v2 and v3') and will only be noticed by a careful reader. Further, it may not be obvious that some analyses were based on only a small number of patients and a handful of events.

Reporting of estimated effects from models and estimates of survival curves often concentrate on DFS and results from OS are less prominently shown. One reason may be the larger number of DFS events, even though OS may be the more important outcome. Reporting the number of deaths may reveal that the effective sample size is very small. To assess the value of any analysis it is important to know both the number of patients and events (the effective sample size) for the outcome.

We attempt to illustrate the issues described above in relation to two rather different studies. The study by Pfisterer *et al*. [157] investigated the effect of ploidy in advanced ovarian cancer (see Table 2). As the disease has a very bad prognosis, the authors decided to consider OS as the only outcome of interest. Part (a) presents the information about the patients, treatments and variables studied. Part (b) gives an overview of all analyses with numbers of patients and events, and the reader is guided to where those results are presented in the report.

The study by Wadehra *et al*. [158] investigated the expression of epithelial membrane protein-2 in patients with endometrial adenocarcinoma (Table 3). In contrast to the first example, both DFS and OS were investigated. Several features are immediately apparent: the sample sizes for these two outcomes differ, only one multivariable analysis was reported for each of the two outcomes, and the marker of interest did not enter the final model for OS. The profile thus gives reviewers, editors and readers a greater opportunity to evaluate what was done and whether anything important is missing. Indeed, creating such a profile should be helpful to authors too.

**Table 3:** Example of the REMARK profile illustrated using data from a study of expression of epithelial membrane protein-2 in patients with endometrial adenocarcinoma [158].

a) Patients, treatment and variables						
**136 **Patients with endometrial adenocarcinoma assessed for eligibility, 37 excluded (33 no informative immune histochemistry, 4 without clinical information)						
**99 **Patients included, stages IA to IVB						
Formalin fixed, paraffin embedded endometrial tissue samples, Department of Pathology, UCLA Los Angeles, USA						

Marker (and how was the marker handled in analysis?)	M = epithelial membrane protein-2 Immunoreactive score obtained by multiplying subscores for intensity (0 to 3+) and distribution of immunoreactivity (0 to 4+) grouped as negative (score 0), weak (1 to 3) or moderate-to-strong (4 to 12)					
Outcomes:	DFS (97 patients, 42 events), OS (99 patients, 32 events)					
Further variables:	v1 = age, v2 = ER, v3 = PR, v4 = vascular invasion, v5 = stage, v6 = histology, v7 = grade					

**b) Statistical analyses of survival outcomes**						

	**DFS**		**OS**			
**Aim**	**Patients**	**Events**	**Patients**	**Events**	**Variables considered**	**Results/remarks**

A1: Univariable	97	42	99	32	M, v1-v7	Figure 3, Figure 4, Table 2, Table 3
						DFS: except v1 all significant
						OS: all significant
A2: Multivariable	97	42	99	32	DFS: M, v2-v7	Table 4, Table 5
					OS: M, v1-v7	In multivariable analysis: all significant in A1, then stepwise selection
						Variables in final models: DFS: M, v5, v6; OS: v4, v6, v7 (M is not included)

DFS: disease-free survival; ER: estrogen receptor; M: epithelial protein; PR: progesterone receptor; OS: overall survival.

Because of the large variety of analyses that may be performed, the profile for a specific study may need to differ in structure from these examples. However, we propose that the key elements of the profile, as shown in the two examples, be included. Wide adoption of this presentation format would considerably aid the transparent reporting of prognostic marker research and help to remedy the widespread deficiencies that have been well documented.

The need for a study profile is supported by the difficulty we encountered in finding published articles that presented all the information to construct a profile. Also, a review of 50 articles in cancer journals in 2006 to 2007 reporting tumor marker prognostic studies found that typically only half of the REMARK profile items were reported and these were often difficult to find [20]. Half of the articles did not report the number of events for any analyses or outcomes.

#### Item 13. Report distributions of basic demographic characteristics (at least age and sex), standard (disease-specific) prognostic variables, and tumor marker, including numbers of missing values

##### Examples

**Figure 2:**
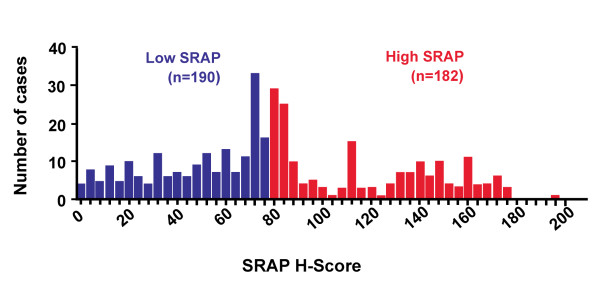
**Frequency distribution of Steroid Receptor RNA Activator Protein (SRAP) H-scores in 372 breast tumors, showing median of 76.67 used to delineate low and high subgroups **[179]** (for a secondary example see Figure 1 in **[29]**)**.

See Table 4, Figure 2 and Figure 1 in [29]

**Table 4:** Example of tabular reporting of patient characteristics [180].

				Patients						
		
	All			*CK-19 *mRNA +			*CK-19 *mRNA -			
Characteristic	Number		%	Number		%	Number		%	*P*
Patients enrolled	444		100	181		40.8	263		59.2	
Age, years										
Median		54			54			55		
Range		26 to 78			26 to 74			30 to 78		0.752
Menopausal status										0.075
Premenopausal	191		43	87		45.5	104		54.5	
Postmenopausal	253		57	94		37.2	159		62.8	
Tumor size										0.648
T1	157		35.4	61		38.9	96		61.1	
T2	251		56.5	103		41	148		59	
T3	36		8.1	17		47.2	19		52.8	
Histology grade										0.316
I/II	204		46	87		42.6	117		57.4	
III	191		43	72		37.7	119		62.3	
Unknown	49		11	22			27			
Infiltrated axillary lymph nodes										0.538
0	163		36.7	61		37.4	102		62.6	
1 to 3	122		27.5	53		43.5	69		56.5	
≥4	159		35.8	67		42.1	92		57.9	
ER										0.779
Negative	175		39.4	71		40.6	104		59.4	
Positive	260		58.6	109		41.9	151		58.1	
Unknown	9		2	1			8			
PR										0.126
Negative	234		52.7	89		38	145		62	
Positive	201		45.3	91		45.3	110		54.7	
Unknown	9		2	1			8			
HER2										0.897
0, 1+	290		65.3	122		42.1	168		57.9	
2+	53		11.9	21		39.6	32		60.4	
3+ by IHC	88		19.8	35		39.8	53		60.2	
Unknown	13		3	3			10			
Adjuvant chemotherapy										0.425
CMF	43		9.7	14		32.6	29		67.4	
FEC	209		47.1	84		40.2	125		59.8	
EC-T	192		43.2	83		43.2	109		56.8	
Surgery										0.478
L	310		69.8	123		39.7	187		60.3	
M	134		30.2	58		43.3	76		56.7	
Radiotherapy										0.799
No	81		18.2	32		39.5	49		60.5	
Yes	363		81.8	149		41	214		59	

CK-19: cytokeratin-19; CMF: cyclophosphamide, methotrexate, fluorouracil; EC-T: epirubicin, cyclophosphamide, docetaxel; ER: estrogen receptor; FEC: fluorouracil, epirubicin, cyclophosphamide; IHC: immunohistochemistry; L: lumpectomy; M: mastectomy; PR: progesterone receptor.

##### Explanation

Inclusion and exclusion criteria (Item 2) describe the target patient population. The group of patients included in a particular study is a sample from that population. Distributions of basic demographic variables and standard prognostic variables should be reported to characterize the group of patients who were actually studied. These demographic and standard prognostic variables are often the variables to be considered for inclusion in multivariable analyses (see Item 8). Distributions of age and sex should routinely be reported. If available, racial or ethnic distributions are sometimes helpful to report, as some markers have shown association with race and/or ethnicity (for example, the positive association between epidermal growth factor receptor gene mutation and Asian ethnicity). For most types of cancers, there are some standard clinical and pathologic prognostic variables (for example, pathologic stage information including nodal status, tumor size and presence of metastases, or clinical measures such as performance status), and distributions of these variables should be reported. The number of patients with missing values should be reported for each variable as should the number of patients for whom there are complete data on all variables or on those variables whose effect on a survival outcome is assessed in a multivariable model.

If patients are a subsample from a randomized trial or large defined cohort it is helpful to compare the characteristics of those with and without tumor marker measurements to help judge the generalizability of the findings.

A thorough description of the distribution of the marker of interest should also be provided. The distribution may be described by a frequency table or bar chart for categorical variables or numerically by use of summary statistics such as mean, median, percentiles, range and standard deviation for continuous variables. Figures such as histograms or boxplots are informative for continuous variables. Presenting continuous data only in categories is insufficient (see Box 4), but grouped data can be presented in addition to the summary statistics.

### Analysis and presentation

#### Item 14. Show the relation of the marker to standard prognostic variables

##### Examples

See Tables 5, 6 and 7.

**Table 5:** Relation between marker (serum chromogranin A) and patient characteristics [181] (note that missing data were not indicated).

	Serum CgA levels, ng/mL				
		
	Number	Median	Q1 to Q3	Minimum to maximum	*P*
**Subjects**					
Controls	50	77.4	57.7 to 99.9	28.2 to 196.3	
NSCLC patients	88	70.4	37.9 to 114.6	8.7 to 723.8	0.337
					
**Histotype**					
Adenocarcinoma	22	59.2	35.2 to 85.6	14.8 to 151.2	
Squamous	27	80.0	41.0 to 128.6	14.7 to 386.8	
Large cell	10	82.1	33.7 to 124.0	11.4 to 217.9	0.465
					
**ECOG PS**					
0	16	37.7	27.2 to 68.6	8.7 to 103.1	
1	59	76.3	43.6 to 119.2	13.9 to 429.7	
≥2	13	102.8	55.8 to 259.4	32.1 to 723.8	0.0005
					
**Stage**					
IIIB	29	44.9	29.2 to 85.6	13.9 to 259.4	
IV	59	82.5	47.1 to 119.2	8.7 to 723.8	0.043

CgA: chromogranin A; ECOG PS: Eastern Cooperative Oncology Group performance status; NSCLC: non-small cell lung cancer; Q1 to Q3: interquartile range.

**Table 6:** Relation between marker (E-Cadherin) and patient characteristics [182].

	E-Cadherin staining index^a^			
	Low		High	
		
Variable	Number of patients	%	Number of patients	%
Histologic type				
Endometrioid^b^	135	53	120	47
Clear-cell or serous papillary	24	83	5	17
FIGO grade				
1	25	49	26	51
2	63	51	61	49
3	71	65	38	35
Vascular invasion				
0 or 1 vessel	94	52	88	48
≥2 vessels	65	64	37	36
Myometrial infiltration^c^, %				
<50	77	51	74	49
≥50	67	66	35	34
FIGO stage^d^				
I or II	120	53	108	47
III or IV	39	71	16	29

^a^Results available in 284 patients; ^b^Adenosquamous and adenoacanthoma are included; ^c^Information available in 253 patients (E-cadherin) and 255 patients (beta-catenin); ^d^Data missing in one patient.

**Table 7:** Relation between patient characteristics and steroid receptor status by immunocytochemistry and dextran-coated charcoal [159]

		Estrogen receptor positive		Progesterone receptor positive	
			
Parameter	n (%)	ICC (%)	DCC (%)	ICC (%)	DCC (%)
Axillary node status (n = 241)					
N0	120 (49.8)	88 (73.3)	98 (81.7)	83 (69.1)	93 (77.5)
N+	121 (50.2)	75 (62.0)	89 (73.6)	75 (61.9)	94 (77.7)
Tumor size (cm) (n = 229^a^)					
<2	86 (37.6)	59 (68.6)	69 (80.2)	60 (69.8)	69 (80.2)
2-5	128 (55.9)	88 (68.8)	101 (78.9)	84 (65.6)	100 (78.1)
>5	15 (6.6)	8 (53.3)	9 (60.0)	8 (53.3)	9 (60.0)
Tumor histology (n = 241)					
Invasive ductal	171 (71.0)	120 (70.2)	136 (79.5)	119 (69.6)	136 (79.5)
Lobular	38 (15.8)	26 (68.4)	31 (81.6)	22 (57.9)	30 (78.9)
Other^b^	32 (13.2)	17 (53.1)	20 (62.5)	17 (53.1)	21 (65.6)
Tumor grade (n = 217)^c^					
1 + 2	142 (65.4)	106 (74.7)	118 (83.1)	104 (73.2)	119 (83.8)
3	75 (34.6)	41 (54.7)	52 (69.3)	36 (48.0)	53 (70.7)
Patient age (y) (n = 241)					
≤50	63 (26.1)	26 (41.3)	42 (66.7)	45 (71.4)	54 (85.7)
>50	178 (73.9)	137 (77.0)	145 (81.5)	113 (63.5)	133 (74.7)

^a^No information available on tumor size in 12 cases; ^b^mucinous, tubular or medullary; ^c^no information available on tumor grade in 24 cases. DCC: dextran-coated charcoal; ICC: immunocytochemistry.

'On analyzing the relationship between receptor data and the above-mentioned prognostic factors, we found a significant correlation between patient age and ER (ICC [immunocytochemistry], r = .46; DCC [dextran-coated charcoal], r = .43). While tumors from patients ≤50 years old were ER positive in only 41% (ICC) and 67% (DCC) of cases, patients >50 years had ER-positive carcinomas in 77% (ICC) and 81% (DCC) of cases. In addition, a weakly significant negative correlation was found between the number of positive axillary nodes and ER (ICC, r = -18; DCC. r = -.15) and a weakly significant negative correlation between tumor grade and ER (ICC, r = -.17) as well as PR (ICC, r = -.24; DCC, r = -.14). No significant correlation between steroid receptors and the remaining prognostic factors, tumor size and histology, was found.' [159]

##### Explanation

The association of the tumor marker with standard prognostic variables should be described. A new marker is most useful if it provides clinically important information beyond that given by existing prognostic variables or indices, or it offers an advantage over other markers because it is easier to measure or quantify. Often a new marker has at least a modest association with some other standard prognostic markers. In a multivariable model, modest correlations between the marker value and other standard variables in the model will influence the estimated effect of the marker and increase its standard error. If there are very strong correlations between two or more variables in a model (for example, between age, estrogen and progesterone receptor in breast cancer), effects estimated from the model can be very unstable and difficult to interpret, requiring great care in model building (see Item 10d). Further, if the marker has a very high correlation with routinely available standard prognostic variables that can be measured more easily, reproducibly and inexpensively, it is unlikely to have clinical value either as a replacement for the standard variables or as an adjunct to the standard variables. Therefore, it is important to report the strength and nature of the association between the marker and other variables. Additionally, it is helpful to summarize the associations between the other standard variables, especially when multivariable models containing combinations of standard variables are being considered.

Graphical displays can be particularly helpful in conveying the nature of associations between the marker and other variables. For two continuous variables (for example, a continuous marker versus a continuous standard variable or prognostic index), scatterplots are most informative, and these may be accompanied by summary measures such as correlations. The study report should include a summary description of the findings of these association assessments. Often the tumor marker and other standard variables are a mix of continuous and categorical measurements. Displays such as boxplots, dotplots or histograms of the continuous measures for each of the levels or combinations of the categorical variables can be informative. Categorizing continuous variables should be avoided (see Box 4). If all variables are categorical, tables showing cross-classifications of cases by categories of the marker and categories of each of the standard variables are useful. Such descriptive analyses are also helpful for interpretation of multivariable models and assessment of the stability of those models.

In order for a marker to provide some information independent of the values of existing variables, it must show variation when the other variables are held fixed. That variation can take different forms. The marker might show variation within all possible ranges of the existing variables, or it might show variation within some ranges of existing variables but not within others. This information, together with an assessment of how the variation in the marker correlates with clinical outcome (see Items 15-17), will suggest those patients for whom the new marker might provide clinically useful new information.

#### Item 15. Present univariable analyses showing the relation between the marker and outcome, with the estimated effect (for example, hazard ratio and survival probability). Preferably provide similar analyses for all other variables being analyzed. For the effect of a tumor marker on a time-to-event outcome, a Kaplan-Meier plot is recommended

##### Examples

**Figure 3:**
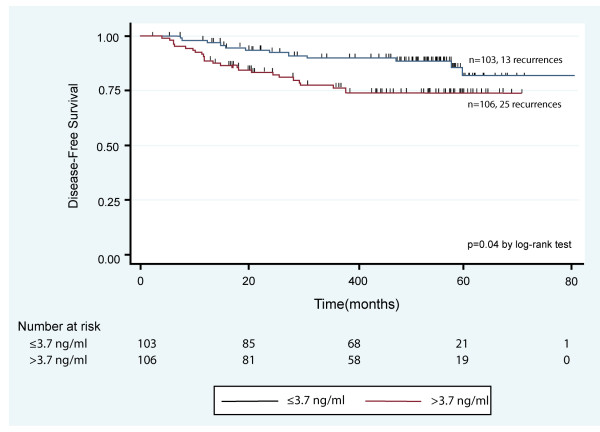
**Kaplan-Meier plot for disease-free survival comparing patients with HU177 concentrations above and below the median value**. [178].

See Figure 3 and Table 8.

**Table 8:** Univariable analyses of relation of UBE2C protein and standard variables to overall survival in 92 women with node-positive breast cancer [183].

Variable	HR	95% CI	*P *
Age	1.06	1.01 to 1.12	0.026
Histology (IDC versus others)	0.48	0.18 to 1.27	0.139
Histological size (<20 mm versus ≥20 mm)	2.97	0.68 to 12.94	0.147
SBR (I versus II versus III)	3.97	1.67 to 9.47	0.001
Positive nodes (1 versus 2 versus 3 versus >3)	1.81	1.19 to 274	0.005
Estrogen receptor (+versus -)	0.18	0.07 to 0.47	<0.001
Progesterone receptor (+versus -)	0.51	0.19 to 1.37	0.182
IHC Ki-67 (<11% versus ≥11%)	8.59	1.14 to 64.57	0.037
IHC UBE2C (<11% versus ≥11%)	7.14	1.64 to 31.11	0.009
NPI scores (1 versus 2 versus 3)	4.48	1.74 to 11.52	0.002

CI: confidence interval; IDC: infiltrating ductal carcinoma; IHC: immunohistochemistry; HR: hazard ratio; NPI: Nottingham Prognostic Index; SBR: Scarff-Bloom-Richardson.

##### Explanation

A marker's simple association with outcome should be shown first, without adjustment for other clinical or pathologic characteristics to indicate its prognostic strength before allowance is made for other variables.

For a binary clinical endpoint (for example, tumor response or disease progression within one year) with a categorical marker, authors can report the observed outcome probabilities for each category of marker value. Sparse categories (those with few patients) may have been combined in the initial data analysis (see Item 10a and Box 4). For a continuous marker it is informative to present a summary of marker values (as in Item 13) separately for those patients with and without the endpoint. Alternatively, a plot of log odds ratio (or a similar measure) as a function of the continuous marker value could be presented. A statistical test of the difference (for example, chi-square test, t-test or test for trend) may accompany the summary description of the association of the marker with the outcome.

For a time-to-event outcome, the relation between a categorical marker and outcome can be assessed by a statistical test such as the logrank test (using the test for trend for ordered categories with more than two groups) [160]. Additionally, a hazard ratio estimate (for example, as derived from a Cox proportional hazards regression model) or some other summary estimate of the association of the marker with survival time should be presented. Precision and uncertainty of the estimates should be indicated, for example by providing confidence intervals. *P *values may also be presented. For continuous markers, one can investigate the influence of the marker on outcome without having to categorize the marker (see Box 4). If any categorizations or transformations are applied to the marker, these need to be clearly stated in order for an association estimate to be interpretable (see Item 11).

Similar analyses are useful for showing the relation to outcome of all other variables being assessed. Such analyses allow confirmation of expected prognostic relations. Results differing from expectations may point to some problems in the study, such as biased patient selection or measurement techniques. Univariable measures of association with outcome can sometimes be presented conveniently along with the distributions of each variable (see Item 13) in a single table.

For a time-to-event outcome, a plot of Kaplan-Meier survival curves is recommended [161,162], with one curve shown for each category of marker value (two curves for a binary marker). The number of patients at risk should be provided for selected time points. To plot Kaplan-Meier estimates for continuous markers or markers with many categories, the marker values are typically combined into a few groups. For continuous markers, the groups are often constructed to contain equal numbers of patients (for example, based on tertiles or quartiles) or the groups may be defined using cutpoints established in a previous study. Regardless of how the groups are constructed, the rationale should be reported. Choosing groups based on maximizing association with outcome is dangerous (see Item 11 and Box 4). It can also be helpful to report estimates of survival probabilities at a few specific time points of interest along with corresponding confidence intervals (for example, 95%) for each marker category.

Univariable measures of association of the marker with outcome and differences between Kaplan-Meier curves might be heavily influenced by other prognostic variables that are correlated with the marker. However, those analyses are still useful to report as they provide a baseline against which to compare measures of association that are adjusted for other variables (multivariable analysis - see Item 16). For this reason it is helpful to present univariable regression analyses as they allow direct comparison of the unadjusted and adjusted hazard ratios.

#### Item 16. For key multivariable analyses, report estimated effects (for example, hazard ratio) with confidence intervals for the marker and, at least for the final model, all other variables in the model

##### Examples

See Tables 9 and 10.

**Table 9:** Multivariable Cox regression analysis of relapse-free survival in patients with primary breast cancer showing the impact of adding the marker (PMN-E) to a base model of recognized prognostic variables [59].

Factor	HR (95% CI)	*P*
*Base model*		
Age and menopausal status combined		0.005
Age premenopausal^a^	0.68 (0.55 to 0.85)	
Age postmenopausal^a^	0.96 (0.84 to 1.09)	
Post- versus premenopausal	1.83 (1.27 to 2.46)	
Tumor size		<0.001
2 cm to 5 cm versus ≤2 cm	1.69 (1.36 to 2.10)	
>5 cm versus ≤2 cm	2.31 (1.73 to 3.10)	
Nodal status		<0.001
N_1-3 _versus N_0_	1.66 (1.30 to 2.11)	
N_>3 _versus N_0_	2.75 (2.18 to 3.47)	
ER (positive versus negative)^b^	0.87 (0.68 to 1.11)	0.25
PgR (positive versus negative)^b^	0.76 (0.61 to 0.95)	0.02

*Additions to base model*		
+PMN-E (high versus low)^c^	1.45 (1.10 to 1.89)	0.01
+PMN-E (continuous)^d^	1.06 (0.98 to 1.14)	0.13

^a^Age in decades for pre- and postmenopausal patients; ^b^Positive, ≥10 fmol/mg protein; negative <10 ng/mg protein; ^c^High, >36.4 ng/mg protein; low, ≤36.4 ng/mg protein; ^d^Log-transformed variable. CI: confidence interval; ER: estrogen receptor; PgR: progesterone receptor.

**Table 10:** Multivariable Cox regression models of overall survival for subgroups of size of residual postoperative tumor [184].

	No residual postoperative			Residual tumor			Residual tumor		
	tumor			1 mm to 10 mm			>10 mm		
			
Parameter	HR	95% CI	*P*	HR	95% CI	*P*	HR	95% CI	*P*
Age (10 y)	1.24	(1.11 to 1.37)	<0.0001	1.12	(1.03 to 1.21)	0.0068	1.10	(1.02 to 1.18)	0.0103
ECOG 2 versus 0-1	1.78	(1.24 to 2.55)	0.0016	1.47	(1.16 to 1.87)	0.0013	1.22	(1.01 to 1.47)	0.0365
FIGO IIIC-IV versus IIB-IIIB	1.41	(1.13 to 1.75)	0.0024	1.49	(1.20 to 1.85)	0.0003	1.48	(1.16 to 1.90)	0.0019
Grading G2/3 versus G1	2.19	(1.45 to 3.30)	0.0002	1.57	(1.00 to 2.46)	0.0524	1.46	(0.99 to 2.15)	0.0569
Endometrioid versus serous	0.84	(0.61 to 1.16)	0.2867	0.95	(0.69 to 1.30)	0.7328	0.97	(0.73 to 1.29)	0.8355
Mucinous versus serous	1.97	(1.26 to 3.08)	0.0028	2.76	(1.90 to 4.02)	<0.0001	2.29	(1.70 to 3.10)	<0.0001
Ascites, yes versus no	1.92	(1.52 to 2.41)	<0.0001	1.18	(0.96 to 1.45)	0.1178	1.31	(1.10 to 1.56)	0.0023

CI: confidence interval; HR: hazard ratio; ECOG: Eastern Cooperative Oncology Group; FIGO: Fédération Internationale de Gynécologie et d'Obstétrique.

##### Explanation

Since a tumor's biological characteristics are not controllable experimentally like treatment in a RCT, a study examining the prognostic value of a tumor marker is subject to the usual challenges inherent in analysis of observational studies, such as adjustment for the effect of potential confounding factors. Some of these other factors are standard variables that are generally accepted as being related to prognosis while others might be candidate variables that are available but have unknown prognostic significance or uncertain relation to the marker of interest. Any of these variables might be considered for inclusion in multivariable models that are developed during the course of the data analysis (see Items 12 and 17). Certain of these multivariable models are of particular importance and the results associated with these models should be reported in more detail.

Often the multivariable data analysis involves a model building process that begins with what we will designate as the 'full model' and, after several data-dependent modeling steps, may result in identification of a 'final model'. The full model is a model containing all the available candidate variables (see Item 8), often depending on decisions from the initial data analysis step considering missing values, distribution of the variables (for example, collapsing of small categories) and other aspects of the data (see Item 10a). Usually the full model contains too many variables to be readily interpretable, but it may serve as the starting point for variable selection, if done, using a method such as backward elimination (see Item 10d) [66]. The final model, which is a more parsimonious model obtained at the end of the variable selection and modeling process, will provide estimates of adjusted effects that are more interpretable, but the effects may also be biased to appear stronger than they actually are due to the variable selection process that had been used. The 'standardized model' (for explanation see Item 17) is another important multivariable model that should be examined in prognostic studies. However, its components are determined on the basis of clinical and pathologic considerations rather than through data-dependent model building, and hence it is discussed separately. The REMARK profile (see Item 12) illustrates which analyses were performed.

As discussed for univariable models (see Item 15), precision and significance of estimated effects should be indicated by providing confidence intervals and *P *values. At least for the final model these measures should be provided for all variables in the model. If multivariable models are also developed for key patient subgroups (for example, separate models for men and women, see Box 1), effect estimates, confidence intervals and *P *values should be provided for all variables in the main subgroup models. For additional multivariable models that do not differ substantially from the main models reported in detail, it may be sufficient to give effect estimates with confidence intervals for the marker of interest only or to summarize results in simple statements. For example, such models might have been used in sensitivity analyses in which a standard variable was eliminated or in which different assumptions were used (see Items 10g and 18).

In a review of 50 studies published in high impact cancer journals in 2006 to 2007, more than one multivariable analysis was reported in 30 of them (60%) [20]. For the primary marker, an effect estimate with confidence interval from the multivariable model was reported in 84%, but only 66% of the papers presented effect estimates for all variables in the final model.

#### Item 17. Among reported results, provide estimated effects with confidence intervals from an analysis in which the marker and standard prognostic variables are included, regardless of their statistical significance

##### Examples

'When all standard prognostic clinical variables were included as co-variables in a Cox proportional hazards model, there was again no evidence that these two markers were significantly associated with OS (HR = 0.99, 95% CI 0.79-1.25 and P = 0.9 for TS [thymidylate synthase] and HR = 0.98, 95% CI 0.78-1.23 and P = 0.8 for p53).' [76]

See Table 11.

**Table 11:** Prognostic values of several factors in a multivariable analysis of overall survival for 175 patients with ovarian carcinoma Stage III/IV [157].

Factor	HR	95% CI	*P *
Age			
≤60	1.00	---	0.051
>60	1.46	1.00 to 2.13	
Stage			
III	1.00	---	0.33
IV	1.20	0.83 to 1.74	
Grade			
1	1.00	---	0.11
2 + 3	1.62	0.89 to 2.94	
Residual tumor			
≤5 mm	1.00	---	<0.001
>5 mm	3.95	1.86 to 8.37	
Ploidy			
diploid	1.00	---	0.93
aneuploid	0.98	0.67 to 1.44	

n = 175, number of events = 133. CI: confidence interval; HR: hazard ratio.

##### Explanation

For many clinical situations one can identify some standard variables that have previously been demonstrated to have prognostic value and are generally measured for most patients having the particular diagnosis. Although there may be some difference from study to study, there may be a core group of variables that are examined in most studies or are recommended in clinical consensus guidelines. Typical standard variables include disease stage and its constituent elements, such as tumor size and nodal status, and sometimes patient demographic variables such as age or sex. Sometimes these variables are used to determine eligibility for inclusion in a study (see Item 2). It is important to evaluate whether the new marker maintains some association with clinical outcome after accounting for these standard prognostic variables. There should be discussion and explanation of how these standard variables have been selected. Sometimes these variables may already belong to an established multivariable score and this should also be referenced [163].

Evaluation of a marker's effect adjusted for standard variables is generally accomplished by examining what we will call the 'standardized model', which includes the marker of interest as well as all of the standard variables, regardless of their statistical significance. Different treatments may be accounted for by indicator variables or by stratification. Irrespective of what other multivariable models are considered, the results of fitting this standardized model should be explicitly reported as it facilitates the comparison of estimated effects of the marker across studies. This model should be clearly distinguished from other multivariable models that may have been fit during the course of the data analysis (see Item 12), particularly the full model and the final model (see Item 16).

Comparison of the effect estimates from the standardized model to univariable effects (see Item 15) and to effects estimated from other key multivariable analyses (see Item 16) will provide a clearer picture of whether the marker contributes prognostic information beyond that provided by existing variables. Therefore, it is important to present the standardized model including estimated effects for the marker and each of the standard variables and measures of their precision and significance as indicated by confidence intervals and *P *values. When the goal is to build a prognostic model and quantify how a model with standard prognostic variables is improved by incorporating the new marker into the model, a measure such as change in predictive accuracy can be presented [164,165] (see also Item 10d).

#### Item 18. If done, report results of further investigations, such as checking assumptions, sensitivity analyses and internal validation

##### Examples

'Estimated effects were similar in the model without stratification (data not shown). In a sensitivity analysis on the complete case population (128 patients, 29 deaths), number of arteries and angioinvasion were still the strongest prognostic factors.' [166]

'No significant deviation from the proportional-hazard assumption could be found by evaluating an interaction term of the change variables and the logarithm of time. Furthermore, the interaction between the change during the first and the change during the second month was not significant.' [167]

'A more detailed investigation with the multivariable fractional polynomial approach did not reveal any strong indication of a nonlinear effect and selected the same variables.' [136]

##### Explanation

Results of many prognostic studies rely on the validity of the statistical models used in the analysis, and inherent in any model are certain assumptions (for example, proportional hazards, linear effects of covariates and missing data mechanisms). Prognostic analysis results will have greater credibility if arguments can be made that the modeling assumptions are likely to be justifiable or that the results are not unduly sensitive to certain assumptions. The report should mention the results obtained from any additional analyses that were performed or diagnostic plots that were examined for the purpose of checking assumptions or demonstrating robustness of results (see Item 10g and Box 4). It will often be impractical or unnecessary to present detailed findings of these assessments, but a brief summary of the findings should be stated. For example, a statement that a smoothed plot of martingale residuals against a covariate exhibited a linear trend would provide support for inclusion of the covariate as a linear term in a Cox proportional hazards regression model; a statement that covariates were checked for possible time-varying effects in a Cox regression model but no significant effect seemed to be present would provide support for the assumption of proportional hazards. Results of assessments for differential marker effects across subgroups or other types of interactions should be reported (see Box 1). Stability analyses, for example, by using the bootstrap [147,168], and conducting assessments including, but not limited to, those mentioned above (see Item 10g) will provide supporting evidence for the appropriateness of final model(s) that provide the basis for the conclusions of the study [99,133].

In some situations, modeling assumptions cannot be empirically verified, and the only recourse may be to demonstrate by sensitivity analyses whether a reasonable range of alternative assumptions still lead to similar conclusions as those reported for the main analysis. For example, this problem is routinely encountered when applying missing data imputation methods [128,130] (see also Box 2). Because true missing data mechanisms are usually unknown, it is recommended that results of any alternative analyses (including complete case analysis) performed under different assumptions about the missing data mechanism (missing completely at random, missing at random or missing not at random) be reported so that the amount the results would change can be assessed.

If either internal validation analyses or external validation studies have been performed (see Item 10h), the results of those analyses should be described, regardless of the findings. Successful validations greatly improve the chances that the study findings are real.

## Discussion

### 

#### Item 19. Interpret the results in the context of the pre-specified hypotheses and other relevant studies; include a discussion of the limitations of the study

##### Examples

'We evaluated the prognostic significance of three VEGF SNPs in a large cohort of patients with esophageal cancer. In multivariate analysis, we showed that the heterozygous and homozygous variant genotype of VEGF 936C/T conferred an improved OS compared with the homozygous wild-type genotype ... Although this is the first study to evaluate VEGF SNPs in esophageal cancer, two prior gastric cancer studies reported conflicting results ... There are limitations to this study. Although others have correlated these VEGF SNPs with plasma VEGF levels, due to the lack of available tissue samples, we were unable to correlate VEGF genotype with VEGF mRNA or protein expression within tumors ... Secondly, the sample size of 361 is very large for esophageal cancer but is only average for all studies evaluating VEGF polymorphisms and cancer outcomes (median sample size, 413; range, 100-1193). Finally, we used a candidate polymorphism approach, which allows us to compare with studies of other disease sites and focuses on functional variants, but therefore will not evaluate the entirety of polymorphic variation across this gene.' [169]

'Our data demonstrate that COX-2 expression is associated with larger tumors, younger patient age, and generally more aggressive breast cancer. These findings are consistent with several other studies that have shown COX-2 expression to be associated with more aggressive disease. Studies evaluating COX-2 expression as it relates to breast cancer aggressiveness and outcome are summarized in Table 4.' [170]

##### Explanation

The discussion is the appropriate section for authors to interpret the data and suggest further research that might be needed. The section should begin by briefly restating the purpose of the study and recalling any pre-specified hypotheses. A simple summary of the major findings should follow. This allows the reader to assess if the study met its goals and to evaluate the evidence. A clear distinction should be made between conclusions based on pre-specified hypotheses and hypotheses suggested during the course of the data analysis.

The authors should critically evaluate the reported results. This evaluation should include an acknowledgment of any biases or inconsistencies in the data, limitations of the assay methods or limitations of the design or data analysis methods. For example, the study may have been underpowered, it may have been limited to only tumors of sufficiently large size, the assay might be lacking in reproducibility, important standard variables may have not been available (for example, tumor grade in breast cancer) and there may have been a large amount of missing data requiring certain assumptions to be made in the analyses. If there are strong biologically plausible subgroup effects, the discussion should review how the prognostic value of the marker varies across those subgroups. A thorough and open discussion will maximize the value of the study results to the broader community, regardless of whether the study results are as the investigators had hoped at the initiation of their study. This discussion should include the authors' assessment of whether the results of the study are generalizable to other populations not studied in the current report. Any unexpected findings should be identified. Even disappointing or unexpected findings can yield important insights.

Following the summary, there should be a discussion of how the results from the study integrate into the existing body of evidence. It is helpful to include an explanation for the choice of references cited (for example, only large studies or only studies in a similar patient population) to allow the reader to evaluate whether selective citation of references has influenced the interpretation of the results. If a systematic review was conducted, it should be described. (If the review was performed prior to initiation of the study, its description may fit better in Item 1.) Authors should comment on whether the results are consistent with, or differ from, the general tendency in previous studies and offer potential explanations for differences.

#### Item 20. Discuss implications for future research and clinical value

##### Example

'The association of SMAD4 gene inactivation with poorer prognosis and an increased propensity to metastasize has direct clinical implications. Some patients with pancreatic cancer have ''borderline'' resectable tumors - they have resectable pancreatic head cancers that are at high risk for a margin-positive resection. Whereas further work is needed, our results, combined with those previously reported in the literature, suggest that patients with borderline resectable pancreatic cancers and SMAD4 gene inactivation might be spared the risk of surgery because their cancer is more likely to metastasize, whereas patients with borderline resectable pancreatic cancers and intact SMAD4 may benefit from the local control provided by neoadjuvant therapy and surgical resection.' [171]

##### Explanation

The rationale for studying any marker, prognostic or otherwise, is to gain relevant information about the biology of the disease, to find new tools to aid in clinical decision-making or to develop new treatments. Observation of a statistically significant association between a marker and an outcome may be encouraging, but in the long term the difference in outcome should have clinically important implications for patient care. If a prognostic marker does not provide added value to existing prognostic information, it may nevertheless be useful if it can be assessed more easily, at lower cost or measured more reproducibly than markers currently used to provide clinically meaningful information.

In some cases, the results of a study will suggest that a marker has some promise for clinical value, but a firm conclusion cannot be drawn due to insufficient information. It is helpful in the discussion of future research plans to specifically identify information that is still lacking or inadequate. For example, further studies might need to be conducted in expanded patient populations or different patient subsets. Contemporary patient populations diagnosed and staged using updated methods and receiving more modern therapies and supportive care might need to be studied. Further research studies may be required to resolve differences in the performance of the marker noted in the literature. The assay method might need refinement to improve its robustness and accuracy before it is ready to be used in routine clinical settings.

Ultimately, the goal of the research is to provide a tool of clinically meaningful value to improve patient outcomes. The discussion needs to provide a clear understanding of what the current study has achieved toward that goal and what steps remain.

## Final comments

Physicians seek information about tumor markers to inform therapeutic decisions for individual patients. The availability of a marker that can distinguish subsets of patients may also influence the design of clinical trials. In order for information about the utility of tumor markers to be appropriately evaluated, the methods used to study the markers and the results generated must be fully reported. The REMARK recommendations were designed to help authors ensure that reports of their tumor marker studies contain the information that readers need. Good reporting reveals the strengths and weaknesses of a study and facilitates sound interpretation and application of study results. The REMARK recommendations may also aid in planning new studies, and may be helpful for peer reviewers and editors in their evaluation of manuscripts.

It was always our intention to supplement the checklist publication [1-7] with a long explanatory paper, as has been done for CONSORT, STROBE and the Preferred Reporting Items for Systematic Reviews and Meta-Analyses (PRISMA) statement, for example [9-11,172]. Following the same model as those articles, in this paper we have provided extensive discussion of each item in the REMARK checklist, providing the rationale and including illustrative examples of good reporting. Where possible we have referred to relevant empirical evidence from reviews of publications. We have also included several boxes to provide additional discussion of some key aspects of prognostic studies.

Although we have primarily focused on studies of single prognostic markers, most of the recommendations apply equally to other types of prognostic studies, including studies of multiple markers, studies to predict response to treatment and studies to develop prognostic models. The REMARK recommendations offer criteria against which to judge the completeness of reporting of marker studies. We hope that improvements will be seen over time, but as yet reviews have shown that incomplete reporting is regrettably common [15,18,20,21,173]. We believe that the REMARK recommendations should be useful in specialties other than cancer, and there are already examples that this is so [21,174-176].

REMARK is not intended to dictate standards for the quality of research and it should not be used as such. However, it can be a useful tool to help assemble the information needed in order to assess the quality and relevance of research.

Reporting recommendations should change as necessary to reflect new empirical evidence and changes in our understanding of which aspects of research are important. We intend to monitor the literature for new evidence and critical comments in the expectation that the checklist will be updated in the future.

Several cancer journals ask authors to follow the REMARK recommendations in their instructions to authors; we encourage more journals to follow this example. Up-to-date information on REMARK and numerous other reporting guidelines can be found on the website of the EQUATOR Network http://www.equator-network.org.

## Box 1. Subgroups and interactions: the analysis of joint effects

It is often of interest to consider whether the effect of a marker differs in relation to a baseline variable, which may be categorical or continuous. Categorical variables, such as stage of disease, naturally define subgroups and continuous variables are often categorized by using one or more cutpoints. Investigating whether the marker effect is different (modified) in subgroups is popular. Epidemiologists speak about effect modification; more generally this phenomenon refers to the interaction between two variables.

In the context of randomized trials, one of these variables is the treatment and the other variable defines subgroups of the population. Here the interaction between treatment and the marker indicates whether the marker is predictive of treatment effect (that is, a predictive marker) [185]. This analysis is easiest for a binary marker. Subgroup analyses are often conducted. The interpretation of their results depends critically on whether the subgroup analyses were pre-specified or conducted *post hoc *based on results seen in the data. Subgroup differences are far more convincing when such an effect had been postulated; unanticipated significant effects are more likely to be chance findings and should be interpreted as being interesting hypotheses needing confirmation from similar trials. The same principles apply to consideration of subgroups in prognostic marker studies.

Subgroup analyses need to be done properly and interpreted cautiously. It is common practice to calculate separate *P *values for the prognostic effect of the marker in separate subgroups, often followed by an erroneous judgment that the marker has an effect in one subgroup but not in the other. However, a significant effect in one group and a non-significant effect in the other is not sound evidence that the effect of the marker differs by subgroup [186,187]. First, a single test of interaction is required to rigorously assess whether effects are different in subgroups [188]. Interactions between two variables are usually investigated by testing the multiplicative term for significance (for example, in a Cox model). In many studies the sample size is too small to allow the detection of other than very large (and arguably implausible) interaction effects [189]. If the test of interaction is significant, then further evaluation may be required to determine the nature of the interaction, particularly whether it is qualitative (effects in opposite directions) or quantitative (effects in same direction but differing in magnitude). Because of the risk of false positive findings, replication is critical [190].

For continuous variables, categorization is a popular approach, but it has many disadvantages: the results depend on the chosen cutpoints (see Item 11 and Box 4), and it reduces the power to detect associations between marker variables and outcome [191]. The multivariable fractional polynomial interaction approach is an alternative that uses full information from the data and avoids specification of cutpoints. It allows investigation of interactions between a binary and a continuous variable, with or without adjustment for other variables [191,192].

Another approach to assess the effect of treatment in relation to a continuous variable is the Subpopulation Treatment Effect Pattern Plot [193].

Both approaches were developed in the context of randomized trials, but they readily apply to observational prognostic studies investigating the interaction of a continuous marker with a binary or a categorical variable such as sex or stage [110,194].

## Box 2. Missing data

Missing data occur in almost all studies. The most common approach to dealing with missing data is to restrict analyses to individuals with complete data on all variables required for a particular analysis. These complete-case analyses can be biased if individuals with missing data are not typical of the whole sample. Furthermore, a small number of missing values in each of several variables can result in a large number of patients excluded from a multivariable analysis. The smaller sample size leads to a reduction in statistical power.

Imputation, in which each missing value is replaced with an estimated value, is a way to include all patients in the analysis. However, simple forms of imputation (for example, replacing values by the stage-specific mean) are likely to produce standard errors that are too small.

Data are described as missing completely at random (MCAR) if the probability that a specific observation is missing does not depend on the value of any observable variables. Data are missing at random (MAR) if missingness depends only on other observed variables. Data are missing not at random (MNAR) if the probability of being missing depends on unobserved values including possibly the missing value itself.

Small amounts of missing data can be imputed using simple methods, but when multiple variables have missing values, multiple imputation is the most common approach [130,195,196]. Most imputation methods assume data are MAR, but this cannot be proved, and these methods require assuming models for the relationship between missing values and the other observed variables. Use of a separate category indicating missing data has been shown to bias results [195].

The plausibility of assumptions made in missing data analyses is generally unverifiable. When more than minimal amounts of data are imputed it is valuable to present results obtained with imputation alongside those from complete case analyses, and to discuss important differences (Item 18).

## Box 3. Clinical outcomes

It is important to clearly define any endpoints examined (see Item 7). Events typically considered in tumor marker prognostic studies include death due to any cause, death from cancer, distant recurrence, local recurrence, tumor progression, new primary tumor or tumor response to treatment. The clinical endpoint is reached when the event occurs. For death, recurrence, progression and new primary tumor, there is usually interest not only in whether the event occurs (endpoint reached), but also the time elapsed (for example, from the date of surgery or date of randomization in a clinical trial) until it occurs. Time until last evaluation is used for patients without an event (time censored). The clinical outcome is the combination of the attainment or non-attainment of the endpoint and the time elapsed. Such clinical outcomes are referred to as time-to-event outcomes. Commonly examined outcomes in tumor marker prognostic studies are disease-free survival (DFS), distant DFS, and overall survival (OS). Different event types are sometimes combined to define a composite endpoint, for example DFS usually includes any recurrence (local, regional or distant) and death due to any cause. For composite endpoints, the time-to-event is the time elapsed until the first of any of the events comprising the composite endpoint occurs. As recently shown, a majority of articles failed to provide a complete specification of events included in endpoints [197].

Many clinical endpoints do not have standard definitions, although there have been some recent efforts to standardize definitions for some disease sites. The STandardized definitions for Efficacy End Points (STEEP) system [67] proposed standardized endpoint definitions for adjuvant breast cancer trials to address inconsistencies such as the fact that new primary tumors, non-cancer death and *in situ *cancers may or may not be included as events in DFS for breast cancer. Different names may be used interchangeably for one survival time outcome, for example, recurrence-free survival and DFS. Furthermore, there is not always agreement on which endpoint is the most relevant endpoint to consider in a particular disease setting. For example, reliable information about cause of death is sometimes not available, so considering death due to any cause is often preferred. In some situations, for example, in an older patient population with small risk of dying from the cancer, it can be argued that death due to cancer is more relevant because it is expected that many deaths will be unrelated to the cancer and including them in the endpoint could make the estimated prognostic effect of the marker difficult to interpret.

The endpoints to be examined should be decided on the basis of clinical relevance. The results for all endpoints that were examined should be reported regardless of the statistical significance of the findings (see Items 15 to 17 and Box 5). A demonstrated association of a marker with one of these endpoints does not guarantee its association with all of the endpoints. For example, local recurrence may be an indication of insensitivity to local or regional therapy (such as radiation therapy) whereas distant recurrence requires that tumor cells have the ability to metastasize. Different markers may be indicative of these distinct characteristics.

## Box 4. Continuous variables

Many markers are recorded as continuous measurements, but in oncology it is common to convert them into categorical form by using one or more cutpoints (Item 11). Common reasons are to simplify the analysis, to make it easier for clinicians to use marker information in decision making, because the functional form of the influence of a marker is often unknown, and to facilitate graphical presentation (for example, Kaplan-Meier curves). Although categorization is required for issues such as decision making, it has to be stressed that categorization of continuous data is unnecessary for statistical analysis. The perceived advantages of a simpler analysis come at a high cost, as explained below. The same considerations apply to both the marker being studied and other continuous variables.

### Categorization

Categorization allows researchers to avoid strong assumptions about the relationship between the marker and risk. However, this comes at the expense of throwing away information. The information loss is greatest when the marker is dichotomized (two categories).

It is well known that the results of analyses can vary if different cutpoints are used for splitting. Dichotomizing does not introduce bias if the split is at the median or some other pre-specified percentile, as is often done. If, however, the cutpoint is chosen based on multiple analyses of the data, in particular taking the value which produced the smallest *P *value, then the *P *value will be much too small and there is a large risk of a false positive finding [198]. An analysis based on the so-called optimal cutpoint will also heavily overestimate the prognostic effect, although bias correction methods are available [199].

Even with a pre-specified cutpoint, dichotomization is statistically inefficient and is thus strongly discouraged [153,200,201]. Further, prognosis is usually estimated from multivariable models so if cutpoints are needed as an aid in classifying people into distinct risk groups this is best done after modeling [153,202].

Categorizing a continuous variable into three or more groups reduces the loss of information but is rarely done in clinical studies (by contrast to epidemiology). Even so, cutpoints result in a model with step functions which is inadequate to describe a smooth relationship [110].

### Keeping variables continuous

A linear functional relationship is the most popular approach for keeping the continuous nature of the covariate. Often that is an acceptable assumption, but it may be incorrect, leading to a mis-specified final model in which a relevant variable may not be included or in which the assumed functional form differs substantially from the unknown true form.

A check for linearity can be done by investigating possible improvement of fit by allowing some form of nonlinearity. For a long time, quadratic or cubic polynomials were used to model non-linear relationships, but the more general family of fractional polynomial (FP) functions provide a rich class of simple functions which often provide an improved fit [203]. Determination of FP specification and model selection can be done simultaneously with a simple and understandable presentation of results [108,110].

Spline functions are another approach to investigate the functional relationship of a continuous marker [101]. They are extremely flexible, but no procedure for simultaneously selecting variables and functional forms has found wide acceptance. Furthermore, even for a univariable spline model, reporting is usually restricted to the plot of the function because presentation of the parameter estimates is too complicated.

When the full information from continuous variables is used in the analysis, the results can be presented in categories to allow them to be used for tasks such as decision making.

## Box 5. Selective reporting

Publication of the findings of only some of the research that was done in a field will lead to bias when publication choices are made with the knowledge of study findings. Selection is mostly in relation to whether or not results were statistically significant (*P *< 0.05) or show a trend in the favored direction. Selective reporting of studies, or selective reporting of only some analyses within studies, both lead to larger effects being seen in smaller studies, and literature that is biased towards overestimating the prognostic importance of tumor markers [204].

Evidence of biased non-publication of whole studies has been accumulating for many years, but recently research has demonstrated evidence of additional within-study selective reporting [205,206]. Empirical evidence of study publication bias and within-study selective reporting primarily relates to randomized controlled trials, but it is likely to be a major concern for prognostic studies. Publication bias in prognostic studies may be worse as many of these studies are based on retrospective analysis of existing clinical databases. Indeed, there is no indication that a particular marker or marker-related hypothesis has been studied until and unless it is published. A review of 1915 articles on cancer prognostic markers found that less than 1.5% were fully negative, in that they did not present any statistically significant prognostic results [207]. A systematic review of studies of Bcl2 in non-small cell lung cancer revealed that almost all the smaller studies showed a statistically significant relationship between Bcl2 and risk of dying with large hazard ratios, whereas the three large studies were all non-significant and showed much smaller effects [208]. A review of the prognostic importance of TP53 status in head and neck cancer showed clearly that published studies had larger effects than unpublished studies [17,209]. Such studies point to the value of a register of biomarker studies [210].

Possible within-study selective reporting could take several forms. For example, in cancer studies two principal outcomes are time to death (overall survival) and time to recurrence of disease (that is, disease-free survival). Many studies report only one of these outcomes. Although both unadjusted and adjusted results are usually provided, some studies only report unadjusted results [211]; in general they will be larger than adjusted results. Similar concerns relate to selective reporting of only some subgroup analyses performed. Reports should include discussion of all analyses performed and whether they were pre-planned (see Item 12). Often a number of exploratory analyses are conducted. The exploratory nature should be clearly stated. Reasons for these analyses and results can be summarized in a few sentences. A further issue is that some results are only reported partially, for example, solely as 'not significant', preventing that study from contributing to a subsequent meta-analysis.

Problems that can arise from selective reporting are discussed in relation to clinical endpoints, the flow of patients through the study and reporting of events and estimated effects for all variables in Items 7, 12 and 16, respectively. Obviously, selective reporting is an important impediment to reliable assessment of a marker according to evidence based medicine criteria [19,212-214].

## Competing interests

The authors declare that they have no competing interests.

## Authors' contributions

All the authors jointly wrote and agreed the text over many years, including several face-to-face meetings and numerous telephone conference calls. All authors read and approved the final manuscript.

## Pre-publication history

The pre-publication history for this paper can be accessed here:

http://www.biomedcentral.com/1741-7015/10/51/prepub

## Supplementary Material

Additional file 1 **REMARK reporting template**. REMARK checklist for authors to complete to accompany a journal submission of a report of a study investigating a prognostic marker. Click here for file
